# An Umbrella Review of Systematic Reviews of School-Based Nutrition Interventions to Determine Outcomes Used and Their Measurement Tools

**DOI:** 10.1093/nutrit/nuaf144

**Published:** 2025-08-11

**Authors:** Dilara Olgacher, Sarah Aldukair, Mike Clarke, Danielle McCarthy, Jayne V Woodside

**Affiliations:** Centre for Public Health, Queen’s University Belfast, Belfast BT12 6BA, United Kingdom; Centre for Public Health, Queen’s University Belfast, Belfast BT12 6BA, United Kingdom; Centre for Public Health, Queen’s University Belfast, Belfast BT12 6BA, United Kingdom; Institute for Global Food Security, Queen's University Belfast, Belfast BT9 5AG, United Kingdom; Centre for Public Health, Queen’s University Belfast, Belfast BT12 6BA, United Kingdom

**Keywords:** school, nutrition intervention, children, adolescents, outcomes

## Abstract

**Context:**

School-based nutrition interventions have the potential to promote dietary behaviors and other outcomes among children and adolescents. However, inconsistent reporting of outcomes and measurement tools limits evidence synthesis and the identification of effective intervention strategies.

**Objective:**

This umbrella review aimed to synthesize the range of outcomes related to diet, health, well-being, and education reported in systematic reviews of school-based nutrition interventions, along with the measurement tools used. The findings will be used to inform the development of a Core Outcome Set (COS) to guide future evaluations in this field.

**Data Sources:**

We conducted a systematic search across 7 databases (MEDLINE, Embase, Cochrane Database of Systematic Reviews, PsycINFO, CINAHL, Web of Science, and Scopus) to identify systematic reviews published from January 2018 to December 2023.

**Data Extraction:**

Data on outcomes related to diet, health, well-being, and education were extracted from eligible systematic reviews. When available, information on outcome measurement tools was also extracted. Additional details were retrieved from primary studies referenced within systematic reviews as needed.

**Data Analysis:**

Ninety-eight systematic reviews, comprising 965 unique studies, were included. Outcomes were categorized into 4 domains: (1) “diet” (68.8%), subdivided into “dietary intake” and “diet-related cognitive/attitudinal behaviors”; (2) “physical health” (44.8%), subdivided into “body composition” and “clinical/biochemical measures”; (3) “social and emotional well-being and behavior” (9.2%); and (4) “education” (6.4%). Substantial heterogeneity was observed across outcome domains, subdomains, and measurement tools, which presents challenges for evidence synthesis and limits comparability across studies.

**Conclusions:**

The findings of this review underscore the need for a stakeholder-informed, context-aware COS to standardize outcome reporting in school-based nutrition interventions. Such standardization is essential to improve the comparability of research findings, enhance evidence synthesis, and facilitate the translation of evidence into policy and practice. Subsequent phases of this work will involve diverse stakeholder engagement to finalize the COS and recommend appropriate tools for outcome measurement for evaluating school-based nutrition interventions.

**Systematic Review Registration:**

PROSPERO registration No. CRD42022378746.

## INTRODUCTION

Childhood and adolescence are critical periods for the formation of healthy lifestyle habits, including dietary behaviors, which often track into adulthood and influence long-term health and well-being.^1^^,^^2^ Despite the importance of this issue, the 2019 edition of the UNICEF *State of the World’s Children* report underscored the ongoing challenges in child nutrition, notably the prevalent consumption of energy-dense, nutrient-poor foods, such as sugar-sweetened beverages (SSBs), and the inadequate intakes of fruits and vegetables (FV).^3^ Consequently, promoting healthy dietary behaviors early in life has emerged as a global public health priority to support health and well-being throughout the life course.^1^^,^^4^

School-based nutrition interventions offer a strategic opportunity to reach large populations of children from diverse ethnic and socioeconomic backgrounds over extended time periods.^4–8^ These interventions have been widely implemented across various regions to improve diet and health outcomes in children. The findings of previous systematic reviews of school-based nutrition interventions have suggested that multicomponent approaches, targeting the curriculum and school environments and policies and involving active stakeholder engagement, are more likely to enhance diet and health outcomes in this population.^9–11^ However, despite shared objectives, substantial heterogeneity remains in how intervention outcomes of school-based nutrition interventions are measured and reported. Previous studies have often evaluated different outcomes or used diverse tools to assess similar outcomes, thereby limiting comparability and hindering the synthesis of robust, generalizable evidence in systematic reviews.^8^^,^^12^^,^^13^ This variability presents an ongoing challenge to formulating conclusive recommendations regarding which interventions are most effective, for whom, and through which mechanisms.^4^^,^^14^^,^^15^

A core outcome set (COS) is an agreed upon, standardized collection of outcomes that represent the minimum outcomes to be measured and reported in clinical trials within a research field.^16^ The application of a COS in school-based nutrition interventions could ensure the consistent assessment of essential outcomes across studies, thereby aiding in the robustness and comparability of evidence syntheses. This process would, in turn, improve the utility of findings for researchers, practitioners, and policymakers in guiding future research and practice.^15^^,^^16^ The initial phase in COS development, guided by the Core Outcome Measures in Effectiveness Trials (COMET) Initiative (http://www.comet-initiative.org/),^17^ usually involves a literature review to extract outcomes from relevant studies and establish a comprehensive list of outcomes. To our knowledge, a COS does not exist for school-based nutrition interventions, nor has there been any attempt to compile the outcomes reported in this field. We therefore conducted an umbrella review of systematic reviews to determine the range of outcomes related to diet, health, well-being, and education that have been used to evaluate the effectiveness of school-based nutrition interventions among children attending primary and secondary school.

## METHODS

### Protocol

This umbrella review was conducted in accordance with the Preferred Reporting Items for Systematic Review and Meta-Analysis (PRISMA)^18^ and Preferred Reporting Items for Overviews of Reviews (PRIOR)^19^ guidelines. The review protocol was registered with PROSPERO, the International Prospective Register of Systematic Reviews (CRD42022378746).^20^

### Eligibility

Systematic reviews, with or without meta-analyses, were included if they reported on studies of school-based nutrition interventions that evaluated outcomes related to diet, health, well-being, or education; targeted primary or secondary school-aged children; and used any comparative effectiveness study design. Eligible interventions included at least 1 nutrition-related component, such as nutrition education (eg, standalone lessons, integration into other subjects, or experiential learning), school meal programs (eg, school feeding or food supplementation), modifications to the school food environment (eg, availability, accessibility, or promotion of nutritious foods through vending machines or cafeteria offerings), or a combination of these components, provided the intervention was delivered within a school setting. Systematic reviews were also included if the interventions in their primary studies addressed additional lifestyle behaviors (eg, physical activity, sedentary behavior, sleep, alcohol use) alongside nutrition. Systematic reviews of interventions delivered across multiple settings (eg, school, home, community) were eligible if they reported separate results for school-based studies. Systematic reviews were included regardless of whether the primary studies they synthesized reported statistically significant post-intervention outcomes, consistent with our aim to capture the full spectrum of outcomes reported. To maintain relevance to the general school-aged population, interventions that targeted children with specific medical conditions, such as attention deficit hyperactivity disorder [ADHD], autism, epilepsy, type 1 diabetes mellitus, celiac disease, or other chronic conditions, were excluded. To capture the most recent synthesis of primary research, only systematic reviews published in English between January 1, 2018, and December 31, 2023 were included (**Table 1**).

**Table 1.** Inclusion and Exclusion Criteria

Category	Inclusion	Exclusion
Participants	Primary and/or secondary school children aged 5–18 y, depending on educational system of respective countries, and presumably healthy with no major comorbidities	Children not enrolled in primary or secondary schools (eg, preschool or kindergarten children, university students) and with conditions or disease that limits application to general population
Intervention	Interventions with ≥1 nutrition-related component, regardless of whether they also target other lifestyle behaviors (eg, physical activity, sedentary behavior, sleep, alcohol use, smoking, mental health).	Interventions which did not contain nutrition component or observational exposures other than programs or policies implemented
Setting	Interventions delivered in primary and/or secondary school settings	Interventions not delivered in primary or secondary school settings (eg, nursery/preschool, kindergarten, university, community, home, clinic)
Study design	Systematic reviews with or without meta-analysis that evaluated effectiveness of school-based nutrition interventions, using a pre- and post-intervention comparison (baseline to follow-up). Systematic reviews that included observational studies (eg, cohort studies) were eligible if they also reported on intervention studies.	Scoping reviews, realist reviews, critical reviews, narrative reviews, umbrella reviews, commentary or letters to the editor; conference abstracts, primary studies
Outcomes	Systematic reviews which included primary studies that reported changes in diet, health, well-being, and/or education-related outcomes	Outcomes not specified in inclusion criteria
Year range	Reviews published from January 2018 to December 2023, with no year restrictions for the studies included in these reviews	Reviews published before January 2018 or after December 2023
Language	Articles published in English	Articles not published in English

### Search Strategy

A systematic search of 7 electronic databases, including MEDLINE, Embase, Cochrane Database of Systematic Reviews, PsycINFO, CINAHL, Web of Science and Scopus, were undertaken in January 2023 and updated in December 2023. A combination of key words for the participants, intervention, and study design were used to identify systematic reviews limited to the English language and published between January 2018 and December 2023. The search strategy, initially devised for MEDLINE, was adapted for other databases. An information specialist reviewed the search strategy to ensure that the appropriate terms and database filters were applied. Additionally, reference lists of all included review articles were checked to identify additional reviews.

### Study Selection

All articles retrieved from the searches were imported into Endnote bibliographic software and duplicates were removed. The remaining articles were transferred to the Rayyan web application tool, where the titles and abstracts of retrieved articles were independently screened by 2 researchers (D.O. and S.A.) using the inclusion/exclusion criteria described above. Any discrepancies between the 2 researchers were resolved through discussion and consensus or, if necessary, by consulting a third researcher (J.V.W.). To ensure consistency, a random 30% of full texts of articles were assessed in duplicate for eligibility (D.O. and S.A.). The remaining articles were then reviewed by D.O., with consideration given to prior discussions. In cases where full texts were not readily available, the lead author was contacted to request the full text for eligibility assessment.

### Data Extraction

A data extraction form was developed in Microsoft Excel and piloted on a subset of included systematic reviews, with refinements made as needed. Data extraction was subsequently completed by 1 researcher (D.O.) and checked by a second researcher (J.V.W.). Data extracted from systematic reviews included first author, year of publication, objective(s), inclusion criteria (study designs, participant ages, intervention characteristics), number of included articles, follow-up duration, and the geographical locations of the included primary studies. Diet, health, well-being, and education outcomes of primary studies, along with their assessment tools when available, were extracted verbatim from the systematic reviews. The outcomes reported in the systematic reviews depended on their specific scope; thus, not all outcomes assessed in the primary studies were necessarily included in the reviews. When relevant information was unavailable in the systematic reviews, an additional investigation of the primary studies was conducted to capture the full range of outcomes evaluated.

### Data Analysis

Duplicate entries of primary studies identified across multiple systematic reviews were removed. All outcomes of primary studies identified from data extraction were aggregated into a long list of outcomes related to diet, physical health, social and emotional well-being, and behavior and education. Outcomes with potential overlap or similarities in their definitions or themes were reviewed and merged to determine domains and subdomains. Outcomes extracted were accordingly assigned to pre-established outcome domains and subdomains. The frequencies of outcomes within each domain and subdomain were estimated using descriptive statistics. Where available, the tools used to assess outcomes were also extracted. Key characteristics of these tools were retrieved from the primary studies, references to the tools or their original sources, or relevant tool repositories, for example the DAPA measurement toolkit (www.measurement-toolkit.org) or NutriTools (www.nutritools.org), to develop a descriptive summary of the tools used across these studies.

Because the primary objective of this project was to determine the outcomes reported in studies of school-based nutrition interventions, the findings are presented as narrative summaries, and a meta-analysis was not performed. Assessment of the quality and risk of bias of the included systematic reviews was not performed as part of this review, because these factors were deemed not relevant to the purpose of the review. This approach is consistent with some of the available literature on COS development.^15^^,^^21^

## RESULTS

### Search Results

**Figure 1.**
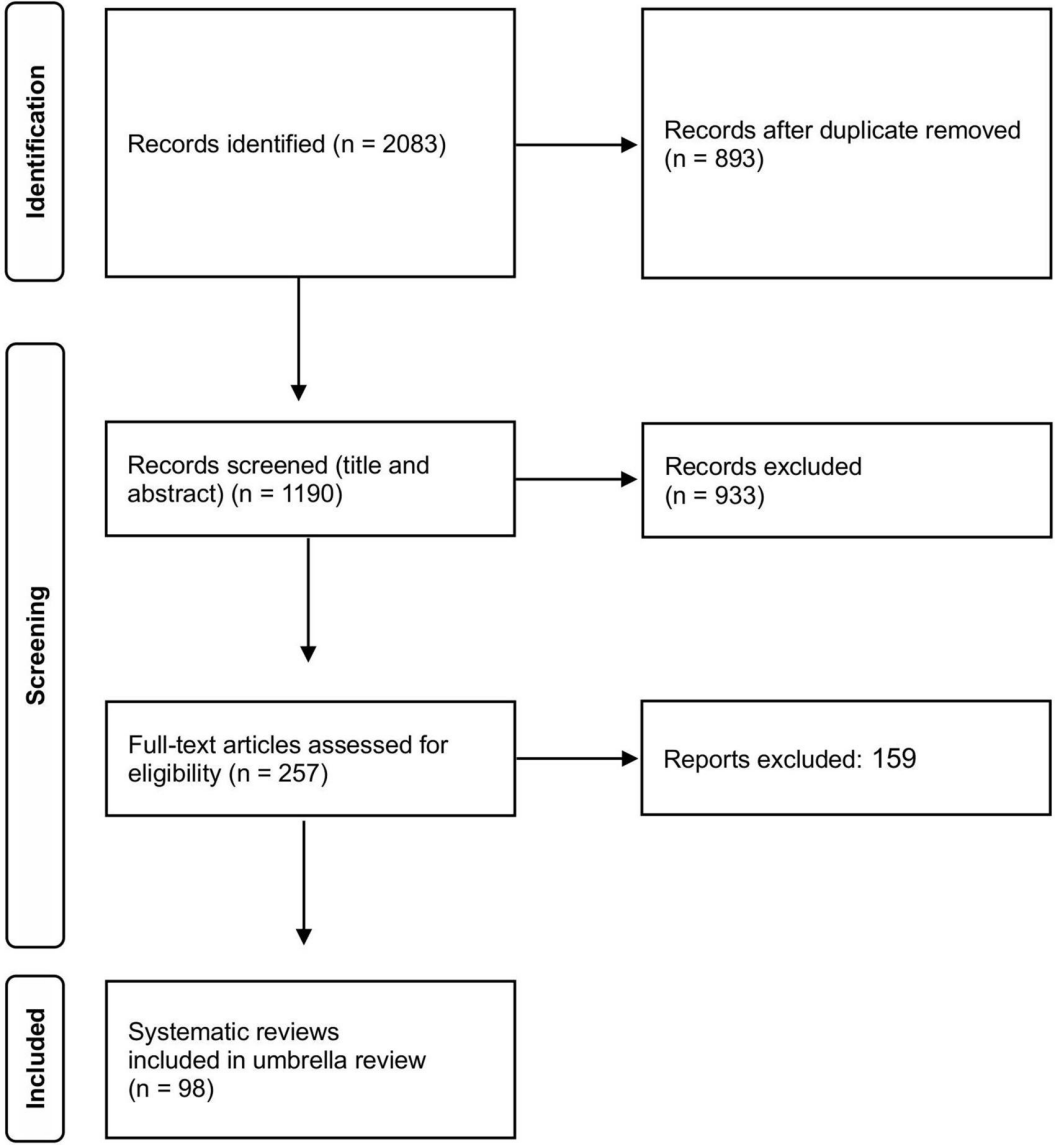
PRISMA Flowchart of Study Selection Process for Studies Included in the Umbrella Review.

The database search identified 1190 unique citations. Titles and abstracts of all articles were screened against the inclusion criteria, and 932 articles were excluded. Following these steps, full texts of the remaining 258 articles were assessed. A total of 98 systematic reviews were eligible for inclusion in this umbrella review. The study selection process is summarized in the PRISMA flow diagram shown in Figure 1.

### Characteristics of the Included Systematic Reviews

Of the 98 systematic reviews included in this umbrella review, the majority either included studies conducted across multiple continents or imposed no geographic restrictions on study inclusion.^4^^,^^6–9^^,^^12–14^^,^^22–82^ A subset of reviews focused on specific regions, including North America, such as Canada,^83^^,^^84^ the United States^85–87^ and Mexico;^88^^,^^89^ Latin America and the Caribbean;^90–92^ Africa;^93–96^ Oceania;^97^^,^^98^ Asia;^10^ and Arab countries.^99^ Three reviews included studies from low- and middle-income countries (LMICs),^100–102^ and 1 review targeted the World Health Organization (WHO) Western Pacific Region, with particular attention focused on LMICs.^103^ Another review included studies from Western high-income countries.^104^ Four reviews included studies that targeted specific population subgroups, including Hispanic children in the United States and Mexico,^105^ American Indian and Alaska Native youth,^106^ Indigenous children,^107^ and racial and ethnic minority groups across multiple regions.^108^ Although schools were the predominant setting for interventions reported in most systematic reviews,^4^^,^^7–10^^,^^12^^,^^14^^,^^25^^,^^27–34^^,^^36^^,^^39^^,^^41^^,^^42^^,^^44^^,^^47^^,^^51–54^^,^^56^^,^^58^^,^^60^^,^^64^^,^^66–68^^,^^70–73^^,^^76^^,^^80^^,^^83–87^^,^^90^^,^^92^^,^^93^^,^^96–99^^,^^101–103^^,^^105^^,^^109^^,^^110^ a substantial number of reviews also included interventions delivered in other settings. These reviews either did not restrict inclusion by setting or reported interventions implemented across multiple environments, including home, clinical, community, and market-based environments.^6^^,^^13^^,^^22–24^^,^^26^^,^^37^^,^^43^^,^^45^^,^^46^^,^^48–50^^,^^55^^,^^57^^,^^59^^,^^61^^,^^62^^,^^65^^,^^69^^,^^74^^,^^75^^,^^77–79^^,^^81^^,^^82^^,^^88^^,^^89^^,^^91^^,^^94^^,^^100^^,^^104^^,^^106–108^ Five reviews reported on interventions delivered via online or digital platforms.^35^^,^^38^^,^^40^^,^^63^^,^^111^ The majority of systematic reviews included studies that focused on populations of school-aged children, including studies focused on primary school children,^9^^,^^10^^,^^12^^,^^14^^,^^27^^,^^29^^,^^31^^,^^33^^,^^34^^,^^56^^,^^58^^,^^60^^,^^64–66^^,^^69^^,^^70^^,^^96^^,^^105^^,^^110^^,^^111^ secondary school children,^22^^,^^24–26^^,^^28^^,^^30^^,^^32^^,^^35^^,^^36^^,^^38–40^^,^^44^^,^^46^^,^^52–54^^,^^57^^,^^62^^,^^67^^,^^68^^,^^71^^,^^73^^,^^76^^,^^79^^,^^80^^,^^97^^,^^98^^,^^101^^,^^103^^,^^108^ or both.^7^^,^^8^^,^^41^^,^^43^^,^^47^^,^^49^^,^^63^^,^^77^^,^^82–87^^,^^90–93^^,^^99^^,^^100^^,^^102^ Several reviews included interventions that targeted children from broader age group ranges (eg, <18 years).^4^^,^^6^^,^^23^^,^^37^^,^^45^^,^^48^^,^^55^^,^^59^^,^^72^^,^^74^^,^^88^^,^^89^^,^^94^^,^^95^^,^^104^^,^^106^^,^^107^ A smaller subset of reviews did not impose any age-related inclusion criteria and reported findings across all age groups, including adults.^13^^,^^42^^,^^50^^,^^51^^,^^61^^,^^75^^,^^78^^,^^81^ In terms of intervention focus, the majority of systematic reviews included nutrition interventions,^4^^,^^8–10^^,^^12^^,^^13^^,^^25–28^^,^^31–34^^,^^37^^,^^39^^,^^41–43^^,^^48–51^^,^^53^^,^^54^^,^^56^^,^^57^^,^^59^^,^^61^^,^^62^^,^^64^^,^^67^^,^^68^^,^^73–75^^,^^77–81^^,^^83^^,^^85–87^^,^^95^^,^^96^^,^^98–102^^,^^106^^,^^111^ whereas others focused on multicomponent interventions that addressed nutrition alongside additional lifestyle behaviors, such as physical activity, sedentary behavior, smoking, alcohol use, sleep, and/or mental health.^6^^,^^7^^,^^14^^,^^22–24^^,^^29^^,^^30^^,^^35^^,^^36^^,^^38^^,^^40^^,^^44–47^^,^^52^^,^^55^^,^^58^^,^^60^^,^^63^^,^^66^^,^^69–72^^,^^82^^,^^84^^,^^88–94^^,^^97^^,^^103–105^^,^^107^^,^^108^^,^^110^ Two systematic reviews that focused on eating disorder interventions^65^^,^^76^ were also included, because they reported studies with a nutritional component. Detailed characteristics of the systematic reviews included in this umbrella review are provided in **Table S1**.

### Outcome Domain Frequency

A total of 965 unique primary studies were identified across the included systematic reviews, with most of these studies appearing in more than 1 systematic review. The outcomes reported in these studies were categorized into 4 domains: (1) diet (with division into 2 subdomains, “dietary intake” and “diet-related cognitive and attitudinal behaviors”); (2) “physical health” (with division into 2 subdomains, “body composition” and “clinical and biochemical measures”); (3) “social and emotional well-being and behavior”; and (4) “education.” A summary of the outcome domains, corresponding subdomains, and illustrative examples used to evaluate the effectiveness of school-based nutrition interventions in school-aged children is provided in **Table 2**.

**Table 2.** Outcome Domains Reported in the Effectiveness of School-Based Nutrition Interventions in School-Aged Children

Outcome domain	Definition	Example of outcomes
1. Diet		
1.1. Dietary intake	Measures of food and beverage consumption, energy and nutrient intake, overall dietary patterns and indicators of food insecurity, hunger or satiety	Food and beverage consumption (eg, fruits and vegetables, sugar sweetened beverages and other food groups) Energy and nutrient intake, including macronutrients (carbohydrates, protein, fats) and micronutrients (vitamins and minerals) Dietary patterns (eg, overall diet quality, dietary diversity, adherence to Mediterranean diet, breakfast consumption, and meal frequency) Food insecurity, hunger or satiety
1.2. Cognitive or attitudinal dietary behaviors	Measures of diet-related cognitive and attitudinal behaviors, including psychological and perceptual aspects that influence food choices and dietary habits	Knowledge and awareness of nutrition, healthy eating, and food-related information Attitudes, beliefs, and perceptions about food, and eating behaviors Food preferences and liking, including specific food items or groups Willingness to try new or unfamiliar foods, including food neophobia and taste acceptance Self-efficacy related to making healthy food choices and maintaining dietary habits Practical skills, such as cooking, food preparation, and gardening
2. Physical health		
2.1. Body composition	Measures of body dimension and composition	Height and weight BMI (as kg/m^2^, *z*-scores, percentiles) Waist circumference Skinfold thickness Weight status Body fat (fat mass, fat‐free mass, android fat mass)
2.2. Clinical and biochemical measures	Measures of cardiovascular, metabolic, endocrine and immunological health as well as indicators of hematological function and nutritional status	Blood lipids (triglycerides, total cholesterol, HDL cholesterol, LDL cholesterol) Metabolic: HOMA‐IR; glucose insulin response; OGTT, HbA1c Blood pressure (systolic blood pressure, diastolic, arterial), heart rate Hematological and nutritional biomarkers
3. Social and emotional well-being and behavior		
Social and emotional well-being and behavior	Measures of the overall state of mental and social well-being, including how individuals feel about themselves, interact with others, and behave in daily life, as well as specific indicators related to disordered eating patterns.	Quality of life, quality of school life Behaviors, knowledge or attitudes related to health and lifestyle factors (eg, lifestyle, sleep, puberty) Emotional and social wellbeing (eg, emotional regulations, mood, self-esteem, self-concept) Mental health (eg, anxiety, stress, depressive symptoms) Eating disorders symptoms (eg, drive for thinness, bulimia and body dissatisfaction, binge eating, media internalization)
5. Education		
Education	Measures of educational factors, including school attendance, academic performance, cognitive abilities, and behavioral indicators within the classroom setting	School attendance or absenteeism Internal efficiency of schools (eg, enrolment, promotion, retention, dropout) Cognitive function (eg, attention, memory, verbal fluency, information processing and abstract reasoning) Academic achievement, measured through subject-specific test scores or aggregate performance Classroom behaviors (eg, attention, participation, hyperactivity, incidents of misconduct, aggression, and disciplinary actions) Curriculum enjoyment

Abbreviations: BMI, body mass index; HbA1c, glycated hemoglobin; HDL, high-density lipoprotein; HOMA‐IR, Homeostasis model assessment for insulin resistance; LDL, low-density lipoprotein; OGTT, oral glucose tolerance test.

### Diet

The most frequently reported outcome domain was diet, which appeared in 68.8% (*n* = 664) of the primary studies included across the systematic reviews. This domain was further divided into 2 subdomains, “dietary intake” and “diet-related cognitive and attitudinal behaviors.”

#### Dietary Intake

Among the primary studies, more than one half (*n* = 558, 57.4%) assessed changes in various aspects of dietary intake. The level of detail in outcome reporting varied across systematic reviews. While some reviews reported broadly on overall diet intake as targeted by the interventions, other reviews offered more detailed analyses of the types and quantities of foods or food groups consumed, such as FVs, grains, dairy products (eg, milk, cheese), protein foods (eg, meat, poultry, fish, eggs), and fats and oils. A number of studies focused on specific food or beverage categories with established links to health outcomes, such as nutrient-dense foods or energy-dense, nutrient-poor items. These included healthy snacks, SSBs, junk food, and savory and sweet snack items. In many cases, study authors assessed a combination of these dietary components of interest. In contrast, in some studies the authors narrowed their focus to single food items, such as water, different types of milk (eg, plain, low fat), apple sauce, orange slices, carrots, green beans, and fish, based on the study aims. For example, Elbel et al. (2015)^112^ and Kenney et al. (2015)^113^ tested the effects of water promotion strategies in school cafeterias, such as the installation of water jets or use of signage and disposable cups, respectively, on student water consumption. In addition to quantifying food-based outcomes, in many studies the authors also quantified the consumption of energy and macronutrients (eg, total fiber, sugars, total fat, saturated fat, cholesterol) and, to a lesser extent, minerals (eg, calcium, folate, iron, sodium, zinc) and vitamins (eg, A, C, and D). In some studies the authors further examined the context in which eating occurred, capturing information on where (eg, at home, at school, in front of the television), when (eg, during recess, at mealtimes, on weekends), and how frequently (eg, eating out ≥3 times per week) foods were consumed. A limited number of studies examined overall dietary patterns and their alignment with dietary guidelines, using indicators such as dietary quality, diet diversity, adherence to Mediterranean Diet foods and eating patterns (eg, breakfast consumption, number of meals per day).

A variety of tools were used to assess changes in dietary intake across the studies. Predominantly, subjective methodologies, such as food frequency questionnaires (FFQs), 24-hour dietary recalls (24HR), food records or diaries, and food checklists, were used to capture the amounts of foods and beverages consumed by children. These tools were administered through various modes, including self-report by children, with or without assistance from parents or caregivers, teachers, trained fieldworkers, or proxies. Studies conducted within the same country frequently utilized the same dietary assessment tools or adapted them from validated tools developed for the respective populations. For example, the Child and Diet Evaluation Tool (CADET) in the United Kingdom (eg, references^114–116^), the Block Food Screener in the United States (eg, references^117–121^), and the Australian Child and Adolescent Eating Survey in Australia were commonly used in their respective contexts (eg, references^122^^,^^123^). These tools were implemented in either paper-based formats or through technology-enhanced platforms, including mobile applications and computer-based software. For example, the Web-based Dietary Assessment Software for Children (WebDASC) was used to assess dietary intake among children in Denmark,^124^ while the Diet-A mobile application captured dietary intake and provided personalized feedback to promote healthier eating among in children in Korea.^125^ Data collection periods reported across systematic reviews varied, with diet recall periods ranging from a single day to 3-7 consecutive days, and data collection was conducted at multiple time points (eg, pre-, mid-, and postintervention). The validation status of the dietary assessment tools was mixed: some tools were validated or adapted from validated instruments while others lacked reported psychometric validation, and, in several cases, validation information was not available within the systematic reviews.

Several studies adopted more objective methodologies to assess food consumption, including digital photography, direct observation, and weighed food diaries. In some cases, multiple dietary assessment tools were used to complement each other. For example, Bontrager et al. (2014)^126^ used a FFQ and lunch tray photo observation to derive FV intakes overall and at school, respectively. Warren et al. (2003)^127^ used a 24HR and an FFQ along with lunch observation to quantify dietary intake. Diet analysis software specified in 2 studies included the Nutrition Data System for Research (NDSR)^128^ and Nutrition Data System (NDS, v4.2).^129^ Biological markers of nutritional status were also measured to identify nutrient adequacy or deficiencies, as further explored above.

Measures of food insecurity, hunger, and/or satiety appeared in a limited number of the studies included in this review. Food insecurity was assessed using parent-reported questionnaires, such as the Community Childhood Hunger Identification Project (CCHIP) scale,^130^ the Food Security Survey Module,^131^^,^^132^ and an 8-item hunger/food insufficiency questionnaire.^133^ Satiety and hunger in children were measured using the “Teddy the Bear” hunger and satiety scale, which allowed children to self-rate their perceived hunger and fullness in relation to both estimated and actual eating episodes. This tool was applied within the context of free school meal interventions^130^ and experiential interventions (eg, workshops in cooking or food shopping).^134^^,^^135^ Further details on the tools used for dietary intake assessment tools, including those related to food security, satiety and hunger, are provided in **Table S2**.

#### Diet-Related Attitudinal or Cognitive-Related Behaviors

A total of 269 primary studies (27.9%) reported the effects of school-based nutrition interventions on a variety of diet-related attitudinal or cognitive outcomes. These outcomes included knowledge and awareness of nutritional topics, such as principles of a balanced diet, the health implications of consumption of specific food items (eg, FVs, water, SSBs, dairy or dairy alternatives, and breakfast), and elements of food literacy (eg, understanding of food labels or nutritional content). Selection and purchasing behaviors were frequently monitored, including choices related to FVs, salads, and vegetarian options, and the nutritional quality of selected meals (eg, energy, saturated fat, sugar, sodium content). Data collection methods for these behaviors included cafeteria point-of-sale records, food service records, direct visual observation by researchers or kitchen staff, tray waste analysis, and digital photography of meal trays. Food preferences and acceptance or unwillingness to taste unfamiliar foods (ie, food neophobia) were also assessed. For example, Chu et al. (2011)^136^ used a 9-point hedonic scale to evaluate children’s acceptance of whole-grain and refined tortillas based on overall liking and sensory attributes, such as taste, color, and softness. Yeo and Edwards (2006),^137^ on the other hand, offered children a different fruit at break time each day for 5 consecutive days and asked them to rank the items in order of their preference to determine fruit preferences.

School-based interventions that integrated experiential learning components, such as cooking, taste tasting, and gardening, evaluated changes in children's knowledge, skills and self-efficacy related to these components (eg, food preparation, planting, or harvesting), as well as their willingness to taste unfamiliar foods, such as new FVs. For example, a school-based kitchen project^138^ was used to evaluated children’s relationships with food, including their enthusiasm for and enjoyment of cooking, alongside improvements in food-related knowledge and awareness. Diet-related attitudinal or cognitive outcomes were often administered through self-report instruments, often in the form of Likert-type questionnaires based on validated tools or developed by study authors to fit the intervention context. Details of the assessment tools used in these studies are provided in **Table S3.** 

### Physical Health

Physical health was the second most frequently reported outcome domain, which appeared in 44.8% of studies (*n* = 432) across the included systematic reviews. This domain was divided into 2 subdomains: “body composition” and “clinical and biochemical measures.”

#### Body Composition

Body mass index (BMI) was one of the primary outcome measures reported in studies included across the systematic reviews. In several reviews, it was noted that weight and height were measured using standardized protocols with calibrated scales and stadiometers, and BMI was subsequently calculated as weight in kilograms divided by the square of height in meters (kg/m^2^). Outcomes were reported as absolute BMI values, *z*-scores, or percentiles, either alone or in combination. In several studies, BMI was also used to classify weight status of children and to evaluate changes in the prevalence of overweight and obesity following the intervention. National or international reference standards were used to interpret BMI in children according to age and sex, including reference standards developed by organizations, such as the Centers for Disease Control and Prevention (CDC) (eg, CDC 2000^139^) and the WHO (eg, WHO 2007^140^), as well as country-specific reference standards (eg, BMI reference curves for the United Kingdom 1990,^114^ age- and sex-specific BMI charts for Germany,^141^ and French reference curves^142^).

In addition to BMI, other measures of adiposity, including waist circumference, body fat percentage, and skinfold thickness (eg, triceps skinfold, mid–upper arm circumference), were reported across the studies. In most cases, the systematic reviews did not provide detailed descriptions of the measurement protocols for these outcomes. However, in one study, waist circumference was measured using a tape positioned at the iliac crest, and in another study, abdominal obesity was defined as a waist circumference above the 90th percentile based on the National Health and Nutrition Examination Survey (NHANES) III reference standards.^140^ Bioelectrical impedance analysis (BIA) and dual-energy X-ray absorptiometry (DEXA) were also used in some studies to estimate fat mass and percentage body fat.

#### Clinical and Biochemical Measures

Several studies assessed cardiometabolic risk factors, often in conjunction with assessments of body composition, as outlined above. The cardiometabolic markers evaluated included measurements of blood pressure (eg, systolic, diastolic, arterial), heart rate, lipid profiles (eg, triglycerides, total cholesterol, high-density lipoprotein [HDL] cholesterol, low-density lipoprotein [LDL] cholesterol), and fasting insulin and glucose concentrations, In addition, assessments of glucose regulation, such as the oral glucose tolerance test (OGTT), and longer-term glycemic control indicators, such as glycated hemoglobin (HbA1c), were also reported. In addition, changes in the prevalence of metabolic syndrome and its associated risk factors were also documented.

Hematological and micronutrient status were assessed in a subset of studies using a range of biochemical measurements. Full blood count analyses were conducted alongside measurements of hemoglobin concentrations, serum or plasma iron levels, transferrin, transferrin saturation, ferritin, and biomarkers of vitamin B12, folate, and riboflavin status. These indicators were commonly used to evaluate the prevalence of anemia. Changes in micronutrient status were further evaluated using a range of biochemical markers, including plasma beta-carotene and serum or plasma retinol for vitamin A, B vitamins, plasma vitamin C, vitamin D, and serum zinc. Urine samples were collected in two studies to assess iodine status and identify deficiencies. Skin carotenoid concentrations were also evaluated in one study. Infection-related outcomes were investigated through physical examinations and laboratory analyses of innate and adaptive immune markers in 1 study, and parasitological outcomes were assessed via fecal sample analysis in another study.

### Social and Emotional Well-Being and Behavior

A range of social and emotional well-being and behavior outcomes were reported in 9.2% of studies (*n* = 89) across the included systematic reviews. Multiple studies measured knowledge related to health topics (eg, puberty, body size and weight, obesity, diabetes, iron deficiency anemia) and lifestyle factors (eg, sedentary behaviors, sleep, smoking, substance use). In some studies, nutrition-related knowledge was also measured alongside broader health and lifestyle topics and was therefore classified within this domain. Constructs related to social and emotional well-being were commonly evaluated, including emotional regulation, self-esteem, self-care, self-efficacy, self-concept, and mindful awareness. Psychosocial outcomes, such as symptoms of depression, anxiety, and mood disturbances, were also assessed, along with quality of life in relation to health and school experiences. In addition, some studies examined variables derived from established behavioral theories and models, including the Health Belief Model^143^ and Social Cognitive Theory.^144^ Most outcomes in this domain were measured using self-report instruments, which were either validated, adapted from validated measures, or developed by study authors. Details of these tools are provided in **Table S4**.

Studies that targeted changes in risk factors related to eating disorders in older children and adolescents assessed changes outcomes related to body image (eg, body esteem, body dissatisfaction, body acceptance, body satisfaction, and weight and shape concern) and disordered eating or extreme weight control behaviors (eg, dietary restraint, emotional eating, or overeating). Internalization of appearance ideals, including cultural and media trends that influence internalization and drive for thinness, was also evaluated. These outcomes were predominantly measured using standardized, validated self-report tools, as detailed in **Table S4**.

### Education

The least frequently reported outcome domain was education, which appeared in 6.4% of the primary studies (*n* = 62) across the included systematic reviews. A range of outcomes related to school attendance, academic performance, cognitive function, and classroom behavior was measured. School attendance was monitored in several studies, with some specifying the criteria applied, such as attendance rates below 95% of school days or recording attendance for morning and afternoon sessions. Academic achievement was evaluated using test scores in specific subjects, including language and literacy, mathematics, science, and social studies, or through aggregated scores across these disciplines. Fewer studies reported on long-term educational attainment or indicators of internal school efficiency, including enrollment rates, grade progression, grade repetition, and dropout rates. Cognitive outcomes were assessed using a variety of neuropsychological tests to examine domains such as attention, memory, verbal fluency, information processing speed, and abstract reasoning. Classroom behavioral and emotional outcomes were addressed in a limited number of studies, in which behaviors such as attention to tasks, interactions, and motor skills were recorded. In one study student enjoyment of the curriculum was assessed using a Likert-type scale. Further details on the tools used for these educational assessments are provided in **Table S5**.

## DISCUSSION

This umbrella review identified substantial heterogeneity, with different frequencies in the outcomes reported across studies of school-based nutrition interventions. Diet-related outcomes were the most frequently assessed, followed by physical health outcomes. In contrast, outcomes related to well-being and education were less commonly reported. This variability underscores the multifaceted nature of nutrition interventions in schools, which can influence a broad spectrum of domains alongside dietary behaviors. Although several systematic reviews conducted meta-analyses on a subset of eligible studies, others were unable to do so due to inconsistencies in outcome selection and measurement methods. This lack of standardization constrains the ability to draw robust conclusions about intervention effectiveness and highlights the need for a COS to guide future evaluations.

The predominance of dietary intake measures reflects our eligibility criteria, which required systematic reviews to include interventions with at least one nutritional component. As a result, dietary behavior outcomes were commonly assessed, regardless of whether nutrition was the central focus of the intervention. A variety of dietary intake outcomes were collected from children and adolescents, including measures of food consumption, nutrient intake, and to a lesser extent, overall dietary patterns. However, the heterogeneity in how these outcomes were defined, measured, and reported limited our ability to synthesize findings, even among studies addressing similar dietary behaviors. These outcomes were commonly measured using subjective dietary assessment tools such as 24HR, FFQ and food diaries. Notably, studies conducted in the same countries often employed similar tools or adapted versions of these validated tools. This consistency was expected, given the need for tools to be appropriate for the target population and to account for cultural context, age, sex, and other determinants of food intake, alongside the study aim, scale, and stakeholder needs.^145^ However, some studies relied on tools with limited or no reported psychometric validation. While these subjective methods offer practical advantages for large-scale evaluations over specific periods, such as being cost- and time-efficient, their accuracy is vulnerable to recall and social desirability biases, potentially leading to under- or overestimation of food intake. These challenges are further compounded by children’s cognitive and memory limitations, which often necessitate proxy reporting.^146^ In contrast, objective dietary assessment methods, such as direct observation, digital photography or nutritional biomarkers, were used less frequently across the studies. These approaches typically provide more accurate or less biased estimates of intake but often cover only a limited number of dietary components. Moreover, objective methods are generally more resource-intensive, may require specialized personnel or equipment, and can be invasive or impractical to implement,^146^ particularly in school settings and at scale. School meal programs often serve as the primary source of regular, nutritious meals, such as breakfast, lunch, or snacks, particularly for students from low-income households who struggle with access to food, thereby contributing to improved diet quality.^32^^,^^96^^,^^102^^,^^147^^,^^148^ Their importance was particularly evident during the COVID-19 pandemic and related school closures.^147^ Nevertheless, the extent to which these programs address broader outcomes related to food insecurity and overall diet quality remains underexplored in the current literature.

The literature on school-based interventions suggests that increased knowledge or attitude do not necessarily translate into desirable changes in food consumption, as behavioral change is a gradual process that necessitates sustained effort and reinforcement over time.^11^ Consequently, the limited duration and intensity of many school-based interventions may constrain the extent to which learners are able to incorporate newly acquired knowledge into their daily routines.^11^ To better capture the trajectory from knowledge acquisition to behavioral change, future evaluations should consider measuring cognitive and attitudinal outcomes alongside behavioral indicators. This approach would provide greater insight into the mechanisms of change and evaluate intervention effectiveness, particularly in the context of longer-term interventions with follow-up assessments.

In relation to physical health outcomes, anthropometric indicators were commonly reported, with BMI being the most frequently used measure. Other body composition indicators, such as waist circumference, skinfold thickness, and body fat percentage were reported less frequently across studies. These additional measures may provide a more comprehensive understanding of body composition and health status, including indicators of undernutrition or overnutrition. Notably, oral health outcomes were absent from the included systematic reviews, despite the well-documented relationship between dietary intake, particularly of free sugars, and dental caries.^149^ This omission aligns with findings from a scoping review by Brown et al. (2021), which reported that oral health was included in only a small proportion of childhood obesity-related interventions. The authors called for greater attention to oral health metrics in future intervention research.^21^ Biochemical and clinical health outcomes were also underrepresented relative to other outcome domains. Although these outcomes can provide information on disease risk, such as such as through lipid profiles, glucose levels, or biomarkers of metabolic syndrome, their assessment in school-based settings may be hindered by ethical, logistical, and financial constraints. These may include the requirement for specialized equipment and trained healthcare personnel, concerns about procedural invasiveness, and the potential for disruption to regular school activities.^145^^,^^150^^,^^151^ Outcomes related to infection control, such as deworming, were largely absent across the included reviews, despite their particular relevance in LMICs. Parasitic infections can impair nutrient absorption and compromise growth and development.^152^ Taken together, these findings underscore the need for future studies to address critical yet often overlooked health outcomes to ensure a more comprehensive evaluation of intervention effectiveness.

Outcomes related to social and emotional well-being and behavior exhibited substantial variability across studies, similar to the diversity observed in diet-related outcomes. These outcomes were assessed using a range of tools, many of which lacked detailed information about their type and validation status. In contrast, the reporting of eating disorder-related outcomes followed a more consistent approach across studies, with the use of standardized instruments widely used in the field that have well-established psychometric properties. School-based nutrition interventions were associated with positive impacts on educational outcomes, such as attendance, concentration, and academic performance, which in turn support students’ ability to learn and thrive^32^^,^^96^^,^^102^^,^^147^^,^^148^^,^^153^ and academic equity,^102^^,^^147^ aligning with the United Nations Sustainable Development Goal 4.^154^ Nonetheless, education-related outcomes remain among the least reported in school-based nutrition research, despite the educational context in which these interventions are delivered and their potential to generate cross-sectoral benefits. This underrepresentation has also been noted in a previous commentary.^155^

The categorization process revealed that many outcomes identified in this umbrella review may span multiple domains. Biomarkers, for example, were classified under the “physical health” domain, as they commonly serve as indicators of health status and disease risk. They can however provide insight into dietary exposure and nutritional status.^156^ Skin carotenoid concentrations, for example, are a non-invasive marker of FV intake,^157^ while levels of iron, folate, and vitamin B12 can identify nutritional causes of anemia.^158^ Similarly, purchasing behavior were categorized under the subdomain of “diet-related cognitive and attitudinal behaviors,” as they are typically driven by individual preferences, knowledge, and decision-making processes. However, these behaviors can also reflect actual consumption patterns and could arguably be placed within the “dietary intake” subdomain. Furthermore, although it was anticipated that severe eating disorder outcomes, such as hospitalization, would fall under the “physical health” domain, no such outcomes were reported in the included reviews. This absence may reflect the scope of our inclusion criteria, for which we focused on the general population rather than those with clinically diagnosed or high-risk conditions. These examples illustrate the interdisciplinary complexity of outcome classification and highlight the need for stakeholder involvement from multiple disciplines in refining COS domains, in order to enhance clarity, consistency, and practical utility.

Schools are important sites for data collection due to their structured routines and access to large, diverse student populations. However, effective data collection in these environments necessitates careful consideration of logistical and contextual factors. Hatch et al. (2023) explored the perspectives of school staff, local authority professionals, and other key stakeholders, on the conduct of health surveys in secondary schools in England. The findings of Hatch et al. underscored the need for research processes to align with the operational demands of school settings. In particular, school staff highlighted that the data collection methods employed by researchers should be flexible, minimally disruptive to the school day, and responsive to teaching and learning priorities, thereby avoiding undue burdens on staff and students.^159^ Technological innovations, such as digital surveys and mobile applications, offer promising avenues to streamline data collection, enhance data quality, and reduce administrative burdens across all stakeholders involved. These tools can also facilitate more efficient data analysis and lower the costs associated with data entry and processing.^21^^,^^145^ Woodside et al. (2021)^155^ similarly advocated innovative strategies to support routine data collection in schools, such as leveraging food purchase data from cashless meal card systems. In this review we further identified examples of digital tools, such as computer software and smartphone applications, used for dietary assessment in children.

### Implications for COS Development

The findings of this umbrella review provide a foundational evidence base for the development of a COS for school-based nutrition interventions. A well-developed COS has the potential to improve the comparability and synthesis of findings across studies, reduce research waste, and inform the development of more effective, evidence-informed interventions and policies.^160^ The next phases of COS development will likely involve a Delphi process to capture stakeholder perspectives on the relative importance of outcomes identified in this review, followed by consensus-building meetings to finalize the COS. Further research will be required to identify validated and reliable measurement tools for each outcome.^17^

To ensure that the COS is applicable across diverse educational contexts, it is critical to consider how outcomes may require prioritization or adaptation in response to contextual factors,^161^ such as school infrastructure, resource availability, cultural norms, and the needs of local stakeholders. For example, while objective or invasive measures (eg, biochemical assays or digital photography of foods) may be feasible in well-resourced schools with access to trained personnel to collect and analyze samples, appropriate technological equipment, and an infrastructure (eg, healthcare professionals and laboratory infrastructure), these measures may be inappropriate or unacceptable in resource-constrained or rural settings, particularly in LMICs. Conversely, outcomes related to hygiene practices and deworming may be of heightened relevance in LMICs, where parasitic infections are more prevalent and pose significant risks to nutritional status, health, and educational attainment,^152^^,^^162^ as previously discussed.

These contextual considerations intersect with methodological features of intervention design, such as study aims, type, intensity, duration, and target population (eg, age, gender).^163^ Given the scope and scale of this umbrella review, which was focused on the outcomes used in the reported studies, such data were not extracted from the primary studies within included reviews. Nevertheless, these methodological factors are likely to influence both the selection of outcomes and the overall effectiveness of interventions.^15^^,^^163^^,^^164^ For example, hands-on, experiential approaches (eg, school gardening, cooking demonstrations) may target behavioral or skill-based outcomes (eg, food preparation skills, vegetable acceptance),^13^^,^^56^ whereas didactic, classroom-based programs may prioritize cognitive outcomes, such as nutrition knowledge or attitudes.^79^

Future syntheses that map outcome selection to intervention strategies or population subgroups could provide valuable insights for the development of a context-sensitive COS. These contextual and methodological considerations can be further addressed in later stages of COS development through the involvement of interdisciplinary stakeholders, including school staff, health professionals, students, caregivers, researchers, and policymakers, from diverse geographic and socioeconomic backgrounds.^164^ The input of these stakeholders will help ensure that prioritized outcomes are not only relevant from a research perspective, but also equitable, locally relevant, and practically feasible. For example, teachers can help identify outcomes that align with both educational objectives (eg, academic achievement and attendance) and the operational capacities of schools.^159^^,^^165^ In addition, we noted limited discussion in the existing literature on the ethical considerations of outcome data collection from children in school settings, particularly in relation to health-related procedures such as blood sampling. To address this gap, we intend to engage professionals with expertise in the ethical conduct of research involving children, who can advise on the selection of appropriate and acceptable measurement tools for ethical integrity. A stakeholder-informed and context-sensitive approach is therefore essential to developing a COS that is both meaningful to end users and implementable across a range of school settings.^16^

### Strengths and Limitations

This umbrella review has several strengths. The methodology was developed in alignment with PRISMA and PRIOR guidelines and conducted according to a published study protocol.^20^ By adopting an umbrella review approach, we were able to identify a large number of systematic reviews on school-based nutrition interventions and synthesize outcomes from over 900 unique primary studies. Although this approach limited our ability to capture the full range of outcomes from reviews with a specific focus, such as those reporting only anthropometric data, we minimized this limitation by directly accessing primary studies when relevant information was not available within the reviews. While minimum outcome sets have been established for evaluations of physical activity interventions in primary schools^164^ and prevention interventions for early childhood obesity,^15^ this review to our knowledge provides the first comprehensive synthesis of diet, physical health, well-being, and educational outcomes from school-based nutrition intervention studies. Outcomes related to physical activity and sedentary behavior were not extracted from the systematic reviews, as these domains have been addressed elsewhere.^164^ This review also has limitations. To ensure feasibility and manageability of data, the search was limited to English-language studies published within the past 5 years. This restriction may have excluded relevant outcomes reported in studies from non–English-speaking regions or older publications. These limitations will be addressed in subsequent phases of COS development, including through stakeholder interviews.

### Conclusion

A wide range of diet, health, well-being, and education-related outcomes have been used in evaluations of school-based nutrition interventions. This variability poses challenges for comparing the findings across studies, thereby complicating evidence synthesis in systematic reviews. The comprehensive list of outcomes identified in this review will inform the next steps in developing a COS for school-based nutrition interventions. The adoption of a stakeholder-informed and context-aware COS across nutrition interventions in schools would standardize outcome reporting, enhance the comparability of findings, and facilitate robust evidence synthesis. Ultimately, such standardization would enhance the translation of research into policy and practice, reduce research waste, and strengthen future intervention strategies.

## Supplementary Material

nuaf144_Supplementary_Data
