# Supplementary material for: An Umbrella Review of Systematic Reviews of School-Based Nutrition Interventions to Determine Outcomes Used and Their Measurement Tools
Source: Nutr Rev. 2025 Aug 11;84(7):1401–17. doi: 10.1093/nutrit/nuaf144 (PMC13253020; doi:10.1093/nutrit/nuaf144)
Supplement: nuaf144_Supplementary_Data [file nuaf144_supplementary_data.zip › Table S1-6.docx]

**Table S1.** Characteristics of systematic reviews included in the umbrella review

| **Author(s), year** | **Objective(s)** | **Inclusion criteria** | | |  | **Number of studies^a^** | **Geographical region of studies** |
| --- | --- | --- | --- | --- | --- | --- | --- |
|  |  | **Design** | **Population** | **Intervention** | **Duration** |  |  |
| Aceves-Martins et al., 2022^1^ | To identify and evaluate studies implemented in Mexico to prevent obesity among children and adolescents | Experimental or QED | Children and adolescents aged <18 years in Mexico | Obesity prevention or lifestyles interventions delivered among Mexican children or adolescents | 2 days to 3 school years | 24/29 | Mexico (29) |
| Aceves-Martins et al., 2022^2^ | To systematically review experimental studies evaluating interventions to treat obesity in Mexican children and adolescents | Experimental studies | Children and adolescents aged <18 years in Mexico | Obesity treatment interventions (through lifestyle, environmental, behavioural, pharmacologic, or surgical interventions) in any setting (e.g., home, school, clinic, community) or digital domains (e.g., mobile-phone-network interventions) | 1 week to 12 months | 5/29 | Mexico (29) |
| Adom et al., 2020^3^ | To characterise and summarise available evidence from school-based interventions that focused on improving nutrition and PA knowledge, attitude, and behaviours, and weight status of learners in the African context. | Controlled or no control study design, with or without randomisation | Children aged 6-15 years in African context | Dietary interventions only, PA interventions only, or combined dietary and PA interventions | 4 months to 3 years | 6/10 | South Africa (6), Tunisia (4) |
| Allcott-Watson et al., 2023^4^ | To identify whether adolescent PA and HE interventions show promise at promoting behaviour change and maintenance, to identify which behaviour change techniques are associated with promising interventions, and to explore the optimal approaches to training deliverers of adolescent PA/HE interventions. | RCT,QED | Children and adolescents aged 10-19 years | Any intervention whose primary aim was to improve PA or HE behaviours including FV consumption | 6 days to 6 months | 5/14 | Turkey (2), UK (1), China (1), Germany (1), USA (2), South Africa (1), New Zealand (1), Iran (2), Netherlands (1), Australia (1), Italy (1) |
| Andrada et al., 2018^5^ | To examine the frameworks used within school-based intervention programs that showed improvements in obesity-related outcomes among Hispanic children in the United States and Mexico | Experimental design | Hispanic children aged 6-12 years with overweight or obesity in the USA and Mexico | School-based intervention programmes that resulted in improvements in obesity-related outcomes (e.g., dietary behaviours and anthropometric measurements) | 10 months to 3 years | 10/10 | Mexico (6), USA (4) |
| Andreo and Andrade., 2020^6^ | To determine effective nutrition intervention strategies and their subsequent impact on nutrition knowledge, dietary adherence, and health outcomes among American Indian/Alaska Native youth | All study designs | American Indian/Alaska Native children and adolescents aged 2-18 years | Nutrition- or diet-related intervention | 3 months to 5 years | 6/12 | USA (12) |
| Andueza et al., 2022^7^ | To evaluate the effectiveness of nutritional interventions carried out in recent years focused on improving the quality of the diet of the child population. | Prospective controlled studies | Children aged 6-12 years | Interventions with a nutritional component focused on diet, school meals, or lunch, aimed at improving diet quality or dietary patterns | 2 months to 3 academic years | 12/12 | Europe (4), United States (6), China (1) and Australia (1) |
| Angawi and Gaissi., 2021^8^ | To evaluate, synthesize, and combine the existing evidence of various setting-based interventions across developed and developing countries that aim to prevent childhood obesity | RCT | Children and adolescents aged 2-19 years | Nonpharmacological interventions for childhood obesity including home-based, school-based, or community-based intervention | 5 weeks to 4 years | 19/32 | Israel (1), France (2), Brazil (2), Australia (2), England (1), China (2), Mexico (1), USA (11), Argentina (1), Chile (1), Spain (4), Canada (1), Switzerland (1), Denmark (1) |
| Bagherniya et al., 2018^9^ | To examine the efficacy of intervention programmes based on social cognitive theory on BMI of adolescents | RCTs, QED | Adolescents aged 12-18 years | Social cognitive theory-based overweight and obesity prevention or treatment interventions, using behavioural strategies to improve dietary habits and/or PA | 12 weeks to 1 year | 5/12 | Australia (4), Brazil (1), Canada (1), USA (6) |
| Bailey et al., 2019^10^ | To synthesise the literature on food literacy interventions among adolescents in secondary schools, the attitudes and perceptions of food literacy interventions in secondary schools, and their effects on dietary outcomes. | Cross-sectional, mixed-methods, quantitative and or qualitative | Adolescents aged 10–19 years | Food literacy interventions with the outcome of a programme related to an improvement in dietary habits, healthy food choices, nutrition knowledge and cooking skills | 1 week to 10 years | 44 | Australia (10), Canada (1), China (1), France (1), Greece (2), Iran (1), South Africa (1), South India (1), Kenya (1), Norway (2), Portugal (1), Denmark (1), USA (17), UK (2), Sweden (2) |
| Bel-Serrat et al., 2022^11^ | To provide a synthesis and critical review of the strategies and theoretical frameworks used in dietary interventions carried out with socioeconomically disadvantaged adolescents living in urban areas. | RCTs, QED, and Pretest-post-test studies | Adolescents aged 12-18 years | Dietary and/or lifestyle interventions, including at least one dietary component | 2 days to 3 years | 38/46 | Australia (4), England (1), Netherlands (1), USA (20) |
| Bennett et al., 2021^12^ | To describe the characteristics of school-based culinary interventions that take place during school hours, and to determine the value of these for child health outcomes. | Not reported | Children aged 5-12 years | Experiential culinary interventions | 4 weeks to one academic year | 5/5 | South Korea (1), USA (3), UK (1) |
| Brown et al., 2019^13^ | To determine the effectiveness of a range of interventions that include diet or PA components, or both, designed to prevent obesity in children. | RCTs | Children aged 0-17 years | Diet or PA interventions, or combined diet and PA interventions, for prevention of overweight or obesity | Minimum 12 weeks | 60/153 | North America (77), Europe (45), Australasia (15), Asia (7), South America (6), the Middle East and North Africa (3) |
| Buru et al., 2020^14^ | To identify the scope of Australian school-based intervention strategies aimed at preventing adolescent obesity and assess the efficacy of the interventions. | Quantitative studies | Adolescents aged 12-18 years old | School-based community interventions for adolescents implemented by school stakeholders. | 4 months to 3 years | 7/13 | Australia (13) |
| Calvert et al., 2019^15^ | To review the types of interventions delivered, dietary behaviours targeted, and interventions' effectiveness in improving dietary behaviour and associated intervention components. | At least one pre‐ and post‐intervention comparison | Children aged 11-16 years old | Dietary behaviour intervention included a component delivered in a school setting (e.g., at lunchtime, during class time, or at before/after school clubs) | 2 weeks to 3 school years | 29/29 | USA (10), Australia (3), Canada (2), England (2), Norway (2), Denmark (2), Greece (1), China (1), Taiwan (1), Israel (1), Belgium (1), Spain (1), Tunisia (1), Netherlands (1) |
| Carducci et al., 2020^16^ | To examine interventions of the food environment and its impact on anthropometric and diet-related health outcomes in school-aged children and adolescents in low- and middle- income countries | RCT, QES, NRCTs, CBA, interrupted-time-series studies, and repeated measures studies | Children and adolescents aged 5-19 years | Preventive and management-based environmental interventions that were implemented at the school (or other organization), community, or market | 3 months to 3 years | 17/17 | China (3), Thailand (2), Mexico (2), Brazil (1), India (1), South Africa (1), Turkey (1), Malaysia (1), Lebanon (1), Tonga (1), Fiji (1), Iran (1), Argentina (1) |
| Cerrato-Carretero et al., 2021^17^ | To identify and perform a meta-analysis on RCTs to evaluate the evidence of the efficacy of long-term school-based interventions in the management of childhood obesity | RCT | Children aged 6-12 years | PA interventions in combination with dietary interventions or dietary education | 18 months to 3.5 years | 11/11 | Norway (1), Spain (1), Iceland (1), Mexico (1), China (2), Chile (1), Germany (3), Australia (1) |
| Champion et al., 2019^18^ | To review the effectiveness of eHealth school-based interventions targeting multiple lifestyle risk behaviours | RCT | Children aged 11–18 years | Universal school-based prevention programme that targeted two or more of the following behaviours: alcohol use, smoking, diet, PA, sedentary behaviour (screen time and sitting), or sleep; and primarily delivered via eHealth methods (e.g., the internet, computers, tablets, mobile technology, or tele-health) | 2 weeks to 36 months | 11/16 | Netherlands (3), USA (11), Belgium (1), Spain and Mexico (1), |
| Chaudhary et al., 2020^19^ | To investigate the evidence for the effectiveness of school-based food and nutrition interventions on health outcomes by reviewing scientific evidence-based intervention studies amongst children at the international level | RCT, CRCT, controlled trial, pre-test/post-test with and without control, experimental design | Children aged 5-14 years | School-based interventions targeting food and nutrition behaviour, healthy eating and nutrition education as a primary focus during the intervention | 1 and half month to 36 months | 42/42 | Europe (26), Asia (6), USA (10), Africa (1) |
| Chavez et al., 2020^20^ | To evaluate the implementation and effectiveness of school-based interventions to prevent obesity conducted in Latin America and provide suggestions for future prevention efforts in countries of the region. | Controlled or before-and-after design | Children and adolescents aged 6-18 years in Latin American countries | Obesity preventions intervention using at least one school-based component and reported obesity-related outcomes | 4 months to two years | 16 | Mexico (5), Chile (4), Brazil (3), Peru (3), Argentina (1) |
| Cohen et al., 2021^21^ | To systematically review the international evidence regarding the impact of universal free school meals on students’ school meal participation rates, diets, attendance, academic performance, and BMI, and school finances. | Not reported | Socioeconomically disadvantaged adolescents aged 12–18 years | Universal school meals | Not reported | 38/47 | USA (25), Denmark (3), Japan (2), Norway (5), Netherlands (1), Greece (2), England (2), Scotland (2), Wales (3), New Zealand (2), |
| Collado-Soler et al., 2023^22^ | To investigate the effectiveness of nutrition intervention programs in children aged 3–12 years worldwide | Experimental or QED | Children aged 3-12 years | Nutrition intervention programmes in pre-primary or primary schools | 4 days to 4 years | 17/19 | USA (5), Malaysia (2), China (2), Spain (1), Norway (1), Australia (1), Italy (1), Canada (1), Tunisia (1), Netherlands (1), Taiwan (1), South Korea (1), Scotland (1) |
| Colley et al., 2019^23^ | To synthesizes current academic literature of school nutrition programmes in Canada to identify existing interventions and their impacts on children’s nutritional knowledge, dietary behaviour, and food intake | RCTs, QED, observational Studies | Children aged 5-18 years | Programmes based in elementary schools that contained a program that offers food provision during the school day, (iv) contained a primary evaluation, assessment, or analysis of the program, and (v) reported a primary outcome that is related to children’s health (e.g., nutritional knowledge, dietary behaviours) | 6 weeks to 4 years | 11 articles (9 studies) | Canada (11) |
| Comeau et al., 2023^24^ | To describe the various nutrition interventions implemented in Australian secondary schools and report their effectiveness in improving nutrition-related health outcomes of students. | Not reported | Adolescents aged 12-18 years | School-based intervention studies that included a nutrition component | 25 days to 4 years | 10/13 | Australia (13) |
| Cotton et al., 2020^25^ | To ascertain the impact of nutrition education programmes on elementary-aged students’ energy intake, FV, sugar consumption and nutritional knowledge | RCT, QED, cluster controlled trial | Elementary-aged children | Teacher-delivered nutrition education programmes | 2 weeks to 6 years | 33/34 | Italy (1), UK (7), USA (13), Netherlands (2), Canada (2), Trinidad & Tobago (1), Ireland (1), Australia (2), Iceland (1) , Greece (1), Portugal (1), Italy (1) |
| Dabravolskaj et al., 2020^26^ | To examine the effectiveness of school-based intervention types perceived by Canadian stakeholders in health and education as feasible, acceptable and sustainable in terms of improving PA, FV intake, and body weight. | Comparative studies | Children aged 4–18 in countries with human development index of 0.80 or greater | School-based interventions to prevent obesity and associated risk factors (i.e., unhealthy diet, PA, sedentary behaviour) | 3 months to 7 years | 52/83 | USA (17), Australia (10), Canada (8), Denmark(7), Spain (7), UK (6), Norway (6),, New Zealand (6), Germany (4), Ireland (2), Italy (2), Switzerland (2), France (2), Belgium (1), Sweden (1), South Korea (1), Israel (1) |
| Dallagiacoma et al., 2023^27^ | To determine the efficacy of digital interventions to promote a healthy diet among children. | Experimental study design | Children aged 5-12 years | Interventions with audio/visual tools aimed at promoting a healthy diet | No follow-up to 3 months | 7/9 | USA (5), Canada (1), Philippines (1), UK (1), China (1) |
| de Sousa et al., 2022^28^ | To review the effectiveness of web-based interventions used in health behavioural change in adolescents regarding PA, eating habits, tobacco and alcohol use, sexual behaviour, and quality of sleep | Experimental studies, QED, before-and-after studies/pre-post-test studies, clinical trials, and RCT | Adolescents aged 10-24 years | Web-based intervention executed using a prescriptive online programme operated through a website and used by consumers seeking health and mental-health related assistance | 3 to 6 months | 1/14 | USA (6), Mexico (2), Taiwan (1) , China (1), Iran (1), UK (2), European countries (Sweden, Germany, Belgium, and the Czech Republic) (1) |
| Dias et al., 2020^29^ | To make a quantitative analysis of the impact of school-based interventions on adolescents’ BMI | RCT | Adolescents aged 12-18 years | School intervention programs reporting BMI change | 8 weeks to 2 years | 10/12 | France (1), Australia (3), USA (4), Netherlands (1), Malaysia (1), Brazil (1), Iran (1) |
| Dimple and Ramesh., 2023^30^ | To explore and critically appraise all the quantitative studies analysing the effect of a cooking intervention on obesity and weight management among children and adolescents | Experimental study design | Children and adolescents aged ≤ 18 y | Meal preparation activity in school/preschool/community (at least 4 sessions during the entire study period) | 2 weeks to 1 year | 4/9 | USA (7), Germany (1), Hungary (1) |
| Egan et al., 2023^31^ | To systematically review the evidence on the effectiveness of eHealth interventions targeting adolescents from disadvantaged backgrounds in preventing poor diet, alcohol use, tobacco smoking, and vaping | RCT and QED | Adolescents aged 10-19 years | eHealth intervention targeting poor diet, alcohol use, tobacco smoking, or vaping | 20 min to 25 months | 6/14 | USA (8), Australia (2), Netherlands (2), Thailand (1), Spain (1) |
| Flores-Vazquez et al., 2023^32^ | To carry out a systematic review of studies among adolescents that implement and evaluate theory-based educational nutrition interventions, in school settings, for the modification of the consumption of food, through controlled trials | Intervention studies with at least one comparison arm | Adolescents aged 10-19 years | Educational nutrition interventions based on theories or models for behaviour change, implemented in school settings, delivered face-to-face and in groups | 2 months to 10 months | 12/12 | India (2), Australia (2), Iran (3), Braxil (1), Turkey (1), USA (2), Greece (1) |
| França et al., 2022^33^ | To provide an overview of the research targeting the effectiveness of mHealth interventions among children and adolescents in the school environment. | Observational and experimental studies | Children and adolescents aged 12-19 years | Interventions that used digital platforms to monitor any type of health condition or to promote health in schools | 4 weeks or less- up to 24 weeks | 2/13 | Not reported |
| Godoy-Cumillaf et al., 2020^34^ | To compare the effect of PA only with that of PA plus diet interventions on BMI in Latin American children and adolescents. | RCT, NRCT, single-arm pre-post study | Children and adolescents aged 4-18 years | PA interventions (physical endurance, sports, or alternative exercise such as games, dancing, optimised physical education classes) including or not including diet intervention | 2 to 36 months | 9/18 | Brazil (5), Mexico (5), Chile (6), Colombia (2) |
| Gordon et al., 2018^35^ | To describe school cafeteria interventions in terms of a behavioural economics scheme and to assess which system is more likely to be effective in improving food selection or consumption | Cross-over and controlled trial design | K-12 | Interventions performed only in the school and cafeteria settings | 1 day to 2 years | 48/48 | Not reported |
| Harris et al., 2021^36^ | To conduct a systematic literature review of the last 20 years of studies on breakfast eating programmes to determine programme effectiveness, analyse their programme features and their use of theory. | RCT | Any participants | Breakfast eating programmes | 48 h to 1 year | 13/19 | USA (6), Canada (1), Australia (5), Iran (1), Turkey (n 1), Israel (1), Gom City (1) and Europe (3) |
| Harrison et al., 2023^37^ | To describe the effects of interventions carried out through public–private partnerships on diet-related obesity risk factors among school-aged children | No restrictions | Children aged 5-18 years | Contact with an intervention, programme, or service that is part of a public–private partnerships | 6 week to 6 month | 3/8 | USA (8) |
| Hasan et al., 2019^38^ | To investigate the effects of culinary interventions on dietary intake and behavioural and cardiometabolic outcomes. | RCT, NRCT, (including cohort and pre-post studies) | Healthy and morbid participants from all ages | Culinary interventions (cooking classes) | 2 weeks to 104 weeks (2 years) | 10/30 | USA (17), Israel (1), Australia (3), UK (3), Japan (2), Ecuador (1), Denmark (1), Kenya (1), Canada (1) |
| Hayba et al., 2020^39^ | To systematically assess the effectiveness of RCTs on lifestyle interventions for the prevention of overweight and obesity in adolescents, with a focus on inclusion of and impact on racial and ethnic minority groups. | RCT | Adolescents aged 13 to 18 years | Interventions with an aim of preventing harmful weight gain and incident overweight and obesity by improving lifestyle factors including nutrition and PA | 15 days to 24 months | 17/30 | USA (10), the Netherlands (3), Australia (6), India (2), Italy (2), Brazil (2), Iran [1], Greece [1], France [1], Sweden [1], Belgium [1) |
| Ho et al., 2021^40^ | To systematically review the impact of school-based intervention for the treatment of childhood obesity. | CRCT | Children and adolescent aged 6-18 years with overweight and obesity | School-based intervention conducted in school or school’s vicinity which aimed to improve nutrition, increase PA, and/or decrease sedentary behaviour | 4 months to 12 months | 9/12 | USA (5), Iran (2), Portugal (1), Germany (1), Thailand (1), India (1), Turkey (1) |
| Ismail et al., 2021^41^ | To examine whether snack-based FV distribution interventions can improve school-aged children’s consumption of FV. | All study designs | Children aged 4-14 years | FV distribution as a snack solely or combined with another intervention approach (e.g. nutrition education, parental involvement) within the school environment | 0.25 months to 12 months | 47/47 | Norway (8), UK (9), Netherlands (4), Multicountry: Norway, Netherlands, and Spain (1), USA(8), Germany (2), Italy (1), Scotland (1), Canada (7), Denmark (2), New Zealand (1), Ireland (1), Australia (1) |
| Jacob et al., 2021^42^ | To synthesize the literature investigating the effectiveness of health education interventions delivered in school settings to prevent overweight and obesity and/or reduce BMI in adolescents, and to explore the key features of effectiveness. | Observational and experimental studies with a control or comparison group | Adolescents aged 10–19 years from high-income countries | Health education interventions delivered in school settings which reported BMI and/or BMI z-scores as outcomes | 3 weeks to | 24/33 | USA (15), Poland (1), Australia (4), Italy (3), Netherlands (4), Norway (1), France (1), UK (1), Greece (1), Spain (1), Sweden (1) |
| Klingberg et al., 2019^43^ | To review existing evidence on the effectiveness of behavioural childhood obesity prevention interventions in African countries on anthropometric and behavioural outcomes | All experimental designs | Children and adolescents aged 2-18 years in African countries | Any behavioural (e.g., PA or diet) interventions aimed at preventing overweight and obesity in any context | 6 days to 3 years | 8/17 | South Africa (n = 9), Tunisia (n = 4), and Uganda (n = 1) |
| Kyere et al., 2020^44^ | To evaluate the effect of school-based nutrition interventions in sub-Saharan Africa on child nutrition status and nutrition-related knowledge, attitudes and behaviour. | RCTs, controlled clinical trials, controlled before-and-after studies or QED with control | Children and adolescents aged 5-19 years | School-based nutrition interventions involving at least one of the following: (a) changes to the school’s physical and social environments; (b) changes to school’s nutrition policies; (c) changes to teaching curricula to incorporate nutrition education and (d) partnership with parents/community. | 3 weeks to 3 years | 14/14 | South Africa(7), Botswana (1), Burkina Faso (1), Kenya (2), Nigeria(2), Tanzania(1) |
| Lavelle et al., 2023^45^ | To synthesize the effectiveness of youth and peer mentor-led intervention programmes on participants and mentors across biometric, nutrition, PA, and psychosocial outcomes | Studies reporting qualitative data | Children and adolescents (K–12) | Youth or peer-led interventions involving mentors in delivery, assessing biometric, nutrition, PA, and psychosocial outcomes in children and adolescents | Not reported | 13/19 | USA (12), Canada (3), UK (4), |
| Leme et al., 2020^46^ | To compare the impact of “energy-balance” and “shared risk factor for obesity and eating disorders” prevention programmes on weight outcome changes; and if the eating disorder risk factors were improved in the “shared risk factor for obesity and eating disorders” programmes. | RCT, NRCT, quasi-experimental control trials, and pre-post uncontrolled studies with no comparison group | Adolescents aged 10-19 years | Energy-balance interventions to improve diet, increase PA, and reduce screen-time with the intent to increase energy expenditure. Shared risk factors for obesity and eating disorders programs as an approach to promote a positive relationship with weight and diet | 4–6 week to 4 year | 13/35 | USA (12), Australia and Oceania (5), Brazil (3), Spain (3), Europe (5) and Asia (3), Canada (1), Mexico (1), Israel (1) |
| Liu et al, 2019^47^ | To assess the overall effects of school-based obesity prevention interventions, and to investigate characteristics of intervention components that are potentially effective for preventing childhood obesity | Individual or cluster-RCT | Children and adolescents aged 5-18 years | Interventions with aim to promote healthy weight or prevention of overweight or obesity rather than treatment of overweight or obesity | 3 months to 6 years | 17/50 | Ireland (1), Switzerland (1), France (1), Spain (5), USA (10), China (5), Italy (1), UK (5), Portugal (1), New Zealand (2), Australia (4), Egypt (1), Brazil (1), Netherlands (2), Greece (2), Germany (1), Iceland (1), Lebanon (1), Mexico (1), Italy (1), Chile (1), India (1), Norway (1) |
| Long et al., 2021^48^ | To determine the effectiveness of dietary interventions on BMI and BMI z-score in childhood | RCT | Children aged <18 years | Medical health education containing dietary contents | 2 to 72 months | 18/28 | Belgium (1), Brazil (1), Chile (1), Crete (1), Denmark, Germany (4), Iceland (1), Iran (1), Malaysia (1), Netherlands (1), Norway (1), Spain (2), Switzerland (1), Sweden, UK (2), USA (7) |
| Lopez-Gil et al., 2023^49^ | To determine the pooled intervention effects of lifestyle-based interventions on improving adherence to the Mediterranean Diet in a young population, and to examine the potential factors related to those intervention effects. | RCT | Children or adolescents aged 3-18 years | Lifestyle-based interventions on adherence to the Mediterranean Diet | 8 weeks to 3 year | 5/12 | Spain (11), Turkey (1) |
| Mandracchia et al., 2021^50^ | To elucidate the effectiveness of full-service restaurant- and canteen-based interventions targeting children, adolescents and adults in increasing the availability, purchase and intake of healthy meals. | Controlled trials, with or without random assignment | Restaurant and canteen consumers (including children and adults) and their staff | Restaurant- and canteen-based interventions concerning the promotion of healthy meals | 1 week to 3 years | 21/41 | USA (20), Australia (11), Lebanon [1], Brazil [1], Denmark [1], Malaysia [1], Scotland [1], Belgium [1], Mexico [1], Ecuador [1], Ireland [1], UK [1]. |
| Marcano-Oliver et al., 2020^51^ | To systematically review the effects of behavioural nudge interventions that modified choice architecture of school canteens at lunchtime, to influence children’s food selection and consumption. | Experimental control, with baseline vs. follow-up comparisons | Not specified | Interventions using only behavioural nudges to promote healthy food item choice or consumption in the school cafeteria. | 2 days to 3 months | 25/25 | USA (24), UK (1) |
| McHugh et al., 2020^52^ | To examine the effectiveness of interventions using the World Health Organization’s Health Promoting Schools framework approach in increasing PA and improving the diet of 11-18-year-olds | CRCT | Adolescents aged 11-18 years | Interventions aimed at changing diet and/or PA levels, which addressed all the components of the World Health Organization’s Health Promoting Schools framework | 5 weeks to 4 years | 9/12 | UnSA (6), Ecuador (1), Belgium (1), Finland (1), France (1), Australia (1), India (1) |
| Medeiros et al., 2022^53^ | To evaluate the effects of school-based food and nutrition education interventions on adolescent food consumption | RCT | Adolescents aged 10-19 years | School-based food and nutrition education interventions on food consumption | 3 weeks to 3 years | 24/24 | USA (9), Iran (2), Greece (2), the Netherlands (2), Italy (1), Norway (1), Brazil (1), Trinidad Tobago (1), UK (1), Belgium (1), Finland (1), Ecuador (1), China (1) |
| Metcalfe et al., 2020^54^ | To determine the range and quality of available evidence of school meal nudges on student eating behaviours, such as school meal participation, food selection, consumption, and waste | Intervention versus post-intervention, control versus intervention, and exposed versus unexposed groups | K-12 | Intervention had to focus on a classic nudge implemented in the cafeteria environment or marketing and promotion campaigns | 1 day to three academic years | 29/29 | USA (26), France (1), Australia (1), and UK (1) |
| Mingay et al., 2022^55^ | To examine nutrition interventions within secondary school dining rooms that provide a routine meal service; the types of intervention strategies implemented, and outcomes measured, and the impact on adolescents’ food behaviours, health and dining experience within this setting | Randomised and non-randomised experimental trials, single group before-after studies | Adolescents aged 10-19 years | Single or multi-strategy nutrition-related interventions that modify the practices of the routine meal service; includes nudging strategies, policy implementation, menu changes, staff training | 1 day to 2 years | 42/42 interventions (35 studies) (7 studies included 2 intervention arms) | USA (31), UK (2), Sweden (1), France (1) |
| Moore et al., 2018^56^ | To evaluate if educational programs can improve understanding and use of nutrition labels | No restriction | Any participants | Interventions which included nutrition label either alone or as a component of a wider multi-component programmes and evaluated outcomes of consumer use and understanding of labels | 10 min to 12 month | 5/17 | USA (12), Canada (2), India (1), Australia (1), UK (1) |
| Morgan et al.,2020^57^ | To assess effects of caregiver involvement in interventions for improving children's dietary intake and physical activity behaviours, including those intended to prevent overweight and obesity | RCTs and quasi-RCTs of parallel group design | Children aged 2-18 years | Interventions to improve children's dietary intake or PA behaviour, or both, with children as active participants and at least one component involving caregivers. | 1 week to 3 years | 15/24 | USA (14), Canada (2), Belgium (3), Germany (1), Australia (1), Iran (1), China (1) |
| Muzaffar et al., 2023^58^ | To compare the gardening, cooking, and combined cooking and gardening programs in elementary schools from the past decade (2011–2022) in improving six psychosocial and behavioural outcomes related to fruit and vegetable intake | Cross-sectional, observational, RCT, randomized controlled factorial, CRCT, QED, and mixed methods including pre/post-tests. | Children aged 5-12 years | Interventions that were primarily garden and/or cooking based in primary schools | 3 weeks to 20 months | 36 | USA (18), UK (4), South Korea (3), Taiwan, South Africa, Belgium, Portugal, Canada, Chile, Netherlands, Slovenia, Australia, Ireland, and Amsterdam |
| Nakabayashi et al., 2020^59^ | To weigh the strength of evidence about the transtheoretical model usage in nutritional interventions for adolescents and its effectiveness regarding dietary intake. | RCTs, QED | Adolescents aged 10-19 years | Nutritional intervention that used at least one transtheoretical model construct in the design | 1 month to 3 years | 11/14 | Brazil (3), USA(5), Turkey (1), Belgium (1), Iran (1), Mexico (1), England (1), Malaysia (1) |
| Nally et al., 2021^60^ | To examine the effectiveness of school-based interventions on primary school children at changing obesity-related behaviours and/or a change in BMI/BMI z-score | RCTs or CRCTs with a comparison or control arm | Children aged 5-12 | Intervention that targeted a change in at least two measures of BMI and/or obesity-related behaviours including PA, sedentary and/or nutrition | 12 weeks to 4 years | 32/38 | UK (5), Iran (1), Greece (3), Mexico (1), Norway (3), Germany (4), USA (8), Ireland (2), Australia (3), New Zealand (1), Lebanon (2), Chile (1), Netherland s (1), China (3), Spain (4), Sweden (1), Canada(1), Argentina (1), Portugal (1), Italy (2) |
| Nathan et al., 2019^61^ | To assess the effectiveness of lunchbox interventions aiming to improve the foods and beverages packed and consumed by children at centre-based care or school; and subsequent impact on children’s adiposity | Parallel comparison groups (e.g., RCTs, controlled clinical trials, non-randomised trials) | Children aged 2-18 years | Interventions that included any educational, experiential, health promotion and/or family or structural or policy or legislative interventions that targeted food provided from home for child consumption during attendance at school or centre-based care (either explicitly or as part of a broader obesity prevention intervention) | 6 weeks to 21 months | 5/10 | USA (3), UK (3), Australia (2), Mexico (1) and Israel (1) |
| Navidad et al., 2021^62^ | To compile the published evidence regarding school interventions at the primary stage aimed at preventing obesity, and which integrate as part of their action plan two features: an improvement in knowledge or nutrition habits and the promotion of PA, and the use of new information and communications technologies to do this | Not reported | Primary schoolchildren | Intervention to improve eating habits or the taking part in PA; used new technologies to carry out said intervention | 9 lessons of 55 min to 4 years | 14/14 | Not reported |
| Nikniaz et al., 2020^63^ | To evaluate community-based interventions for improving dairy consumption and their effectiveness to help policymakers in designing coherent public health strategies | RCTs, QD | All ages | Community-based interventions to increase dairy/calcium consumption | 5 weeks to 350 weeks | 11/25 | USA (16), Japan (2), Australia (1),Canada (3), Iran (1), New Zealand (1) , India (1) |
| Nonguierma et al., 2022^64^ | To conduct a systematic review of randomized controlled trials aimed at improving dietary intake and behaviours among adolescents | RCTs | Adolescents aged 10-19 years | Intervention activities related to dietary behaviours, dietary intake, and dietary habits | 5 weeks to 28 months | 21/36 | Iran (3), Netherlands (1), USA (13), Brazil (2), Turkey (1), South Sulawesi (1), Korea (1), Ecuador (1), New Zealand (1), Australia (4), Denmark (1), Portugal (1), Italy (2) |
| Obita and Alkhatib., 2023^65^ | To examine the effectiveness of lifestyle interventions in children from minority ethnic populations in Western high-income countries | Quasi-randomised studies or RCTs | Children aged <18 years | Lifestyle interventions for the prevention of overweight/obesity and related non communicable diseases | 8 weeks to 5 years | 40/53 | USA (52), UK (1) |
| Oh et al., 2022^66^ | To evaluate the use of digital platforms for universal health promotion in school-aged children and adolescents, which include healthy living, physical activity, nutrition, reduction in screen use, sedentary behaviours, online risk exposure, and mental health globally | RCTs, QED, and nonrandomized trials | Children and adolescents aged 5-19 years | Digital-based interventions that aim to promote healthy lifestyle habits and behaviours, prevent chronic conditions (e.g. overweight and obesity), and reduce leisure screen time and sedentary behaviour | 1 day to 20 months | 32/74 | Australia (8), Belgium (2),Canada (3), France (2), England (3), Germany (2), Hong Kong (3), the Netherlands (3), New Zealand (4), Portugal (2), the United States (21), Denmark (1) Finland (1) Italy (1), Japan (1), South Korea (1), Spain (1), Norway (1),multi country study compromised of high income countries only (1), Brazil (5), Thailand (2), China (2),Ghana (1), Indonesia (1), Malaysia (1), Turkey (1) |
| Olarte et al., 2023^67^ | To systematically review the evidence regarding the impact of Breakfast After The Bell initiatives on students’ nutritional and educational outcomes, including breakfast participation, diet quality and consumption, BMI and weight status, attendance, classroom behaviour, and academic performance. | Quantitative research articles | Elementary, middle and high-school aged students in the USA | Breakfast in the Classroom initiatives conducted in elementary, middle and high schools (public or private, K-12) during the school year | Not reported | 37/37 | USA (37) |
| Omidvar et al., 2022^68^ | To identify: (1) strategies and principal components of Food and Nutrition Literacy promotion, (2) the implementation methods of the interventions, and (3) the effectiveness of interventions in promoting Food and Nutrition Literacy among primary schoolchildren. | RCTs, non-RCTs, quasi-randomised trials, pre- and post-test, post-test only and case–control designs | Children aged 5-12 years | Interventions that contained one or more dimensions of the skill domain of food/nutrition literacy, including functional, interactive and critical food/nutrition literacy that targeted children in elementary schools, or other equivalent educational settings | 5 weeks to 10 month | 19/19 | USA (15), Australia (2), UK (1), Spain (1) |
| Palmer et al., 2023^69^ | To determine the quality and breadth of available evidence of the effectiveness of policy, systems, and environmental change strategies on the consumption and waste of targeted school meal components (fruit, vegetable, milk, and water). | Not reported | K-12 school students in the USA | Interventions incorporating policy, systems, or environmental changes to eating behaviours within the K-12 cafeteria during breakfast | 1 lunch period to 1 year | 23/30 | USA (30) |
| Pineda et al., 2021^70^ | To assess the effectiveness of school food environment interventions in the prevention of childhood obesity, with a focus on adiposity and dietary intake outcomes, based on studies published up to 2020. | RCT, QED | Children aged ≤19 year | Interventions that focus on the school food environment with the aim to shape accessibility, affordability, desirability, and convenience of food acquirement and consumption to prevent obesity or improve dietary intake | 5 weeks to 2 years | 100/100 | USA (53), UK (10), Norway (6), Latin America (6). Other countries represented in the review include China, India, New Zealand, Denmark, Finland, Canada, Australia, Jamaica, Italy, and the Netherlands |
| Pongutta et al., 2022^71^ | To determine the effectiveness of primary school nutrition programmes on reducing any forms of malnutrition among school-aged children in Asian countries | RCT, CRCTs or QED | School-aged children | School-based nutrition interventions for any types of nutritional status implemented in primary schools in Asia | 8 weeks to five school years | 28/28 | China (12), Hong Kong–China (2), Taiwan–China (1), Korea (1), Turkey (3), Lebanon (2), Israel (2), Iran (1), India (1), Malaysia (1), and Thailand (1) |
| Prescott et al., 2020^72^ | To assess the impact of farm to school activities on outcomes of student attending USA schools participating in the National School Lunch Program. | Not reported | K-12 | Farm to school programs and farm to school-related activities | 18 month to 2.5 years | 21/21 | USA (21) |
| Pursey et al., 2021^73^ | To systematically appraise and meta-analyse the effect of universal–selective prevention interventions addressing disordered eating, body image concerns and extreme weight control behaviours in primary school children, as well as moderators such as participant gender and intervention type | RCTs, nonrandomized or quasi-randomized controlled trials, cohort studies and pre–post studies | Children aged 6-12 years | Universal–selective prevention intervention addressing disordered eating, body image concerns and/or extreme weight control behaviours | 1 week to 8 years | 33/39 | United States (17), Australia (5), Canada (4), UK (4), Germany (2) |
| Qi et al., 2021^74^ | To evaluate the effect of school gardening activities combined with PAs on children’s dietary intake and anthropometric outcomes. | RCTs | Children aged 7-12 years | School gardening activities combined and PAs for obesity prevention | 10 to 52 weeks | 14/14 | Not reported |
| Rahman et al., 2018^75^ | To explore the impact of educational and behavioural interventions to reduce the intake of SSBs among children and adolescents across different settings and to assess the effect of these interventions on change in body weight and other health outcomes, taking into consideration which behavioural change techniques were included in these. | RCTs | Children and adolescents aged 4-16 years | Educational or behavioural interventions targeting the reduction of sugar sweetened beverages consumption as one of the main purposes across different settings (e.g., school, home, and community settings) | 10 week to 2 year | 11/16 | Chile (1), Germany (3), Norway (1), Brazil(2), The Netherlands (3), Belgium (1), Portugal (1), USA (3), England (1) |
| Rose et al., 2021^76^ | To explore the effectiveness of school nutrition interventions and the perceptions of young people experiencing a nutrition focused intervention or change in school food policy | No restrictions | Adolescents aged 11-18 years | Nutrition focused interventions or policy approaches in the UK and Europe |  | 22/22 | UK (3), Netherlands (3), Italy (3), Greece (2), Finland (2), France (1), Turkey (2), Spain (3), Norway (1), Portugal (1) |
| Salam et al., 2019^77^ | To assess the impact of preventive nutrition interventions on health and nutritional status of adolescents aged 10-19 years in low- and middle-income countries | Experimental and QED, RCTs, controlled before–after studies, and interrupted time series | Adolescents aged 10-19 years from low- and middle-income countries | Interventions alone or in any combination of nutrition education and counselling, micronutrient supplementation and macronutrient supplementation | 10 weeks to two years | 10/10 | India (5), Indonesia (2), Sri Lanka (1), Bangledesh (1), China (1) |
| Schulte et al., 2023^78^ | To systematically review the impact of choice architecture interventions on the food choice of healthy adolescents in a secondary school setting. | Quantitative studies | Adolescents aged 10-18 years | Choice architecture interventions in a secondary school setting | 4 days to 1 year | 14/14 | USA (11), Denmark (1), Germany (1), UK (1) |
| Seral-Cortes et al., 2021^79^ | To identify the effectiveness in terms of body composition parameters in a generation of articles to prevent obesity and type 2 diabetes in children | RCTs | Children aged 6-12 years | Diet, PA and behavioural support alone or combined with other kind of intervention | 6 weeks to 3.5 years | 12/41 | UK (2), Norway (1), USA (10), Australia (4), New Zealand (5), Netherlands (2), Hungary (1), Denmark(1), China (1), Spain (1), Iceland (1), Israel (3), Sweden (2), Mexico (2), France (1), Finland (1), Hong Kong (1), Switzerland (1), Brazil (1) |
| Shapu et al., 2020^80^ | To determine the effectiveness of health education intervention on improving knowledge, attitudes and practices of adolescents on malnutrition. | RCTs | Adolescents aged 10 to 19 years old | Interventions on nutrition, healthy eating/diet, dietary intake, anaemia, FV | 5 weeks to 2 years | 5/8 | Bangladesh (1), USA (3), China (1), India (1), Canada (1), Palestine (1) |
| Shihab et al., 2023^81^ | To systematically characterize and evaluate the available scientific evidence pertaining to school-based nutrition interventions completed to date across the Arab world. | Experimental intervention studies with quantitative outcomes | School-aged students in any country of the Arab League | Any kind of school-based intervention that addresses nutrition-related aspects | 2 weeks to three years | 15/16 | Lebanon (4), Egypt (2), Tunisia (4), Iraq (1), Palestine (1), United Arab Emirates (1), Jordan (1), Morocco (1), Kuwait (1) |
| Silva et al.,2022^82^ | To systematically assess the effects of computer-based interventions for the promotion of healthy eating on anthropometric indicators and food consumption of adolescents at school | RCTs, QED with a control group | Adolescents aged 10-19 years | Computer-based nutrition interventions using computerized feedback (website and CD-ROM) carried out in the school environment | 1 month to 2 school years | 13/13 | USA (6), Europe (1), Belgium (2), Netherlands (2), Canada (1), Brazil (1) |
| Singhal et al., 2020^83^ | To summarize the evidence from randomized controlled trials evaluating the effectiveness of school-based interventions in preventing childhood obesity in middle-income countries. | RCTs or cluster RCTs | Children aged 4-12 years | Interventions targeting dietary intake and/or PA | 3.5 to 72 months | 15/21 | Brazil (4), China (10), Lebanon (1), Mexico (3), Turkey (1), Iran (1), Thailand (1), |
| Smit et al., 2023^84^ | To investigate the long-term effects of primary school-based obesity prevention interventions on BMI (and z-scores), waist circumference (and z-scores) and weight status | RCTs and studies with other controlled experimental and observational designs | Children aged 6-12 years | Primary school-based obesity prevention interventions, containing at least a diet or PA component | 6 to 36 months | 12/19 | Australia (2), UK (3), Norway (2), Canada (1), China (2), USA (3), Lebanon (1), Slovenia (1), Spain (2), Switzerland (1), Sweden (1) |
| Smith et al., 2021^85^ | To review health literacy-related school-based interventions in adolescents from socioeconomically disadvantaged backgrounds and to identify effective intervention strategies to improve health literacy for this population. | Not specified | Adolescents aged 12-16 years | Intervention related to health literacy in at least one of the following areas: PA, sedentary behaviour, dietary habits, sleeping habits, mental health or substance abuse | 1 session to 1 year | 17/41 | Spain (1), USA (23), Chile (1), Brazil (4), Canada (1), Australia (6), Belgium (1), Sweden (2), India (2) |
| Sutherland et al., 2022^86^ | To assess the effectiveness, when scaled up, of public health nutrition interventions with proven efficacy examined in a randomized controlled trial | RCT, before-and-after trials, and noncontrolled before-and-after designs | Presumably healthy children, adolescents, or adults in nonclinical community settings | Nutrition interventions targeting the prevention of chronic disease and intentionally delivered to a population on a larger scale than the preceding RCT that established the intervention’s efficacy for improving at least 1 dietary outcome | 12 weeks to 2 years | 4/10 | Australia (3), USA (3), Canada (1), UK (1), Netherlands (1), Sweden (1) |
| Taghizadeh et al., 2020^87^ | To systematically search controlled trials that evaluated the effectiveness of paediatric obesity prevention policies among children and adolescents and to analyse the effectiveness of these policies on the study outcomes of body mass index and BMI-Z score measurements while considering a possible dose–response association with preventive tools. | RCT, quasi randomized trials, and CRCT | Children and adolescents aged 0-18 years | Community-based interventions delivered in school (delivered as part of the curriculum or within school hours or after school programmes, changes to school environments/policies (e.g. foods available in the canteen, water fountain installation) Include programs which are primary prevention only Policy changes (e.g. strategies, plans) Environmental changes or interventions—e.g. new parks, water fountain installations Community health service; other community setting (church, sports club, NGO, councils) | 6 weeks to 3 years | 29/64 | United States (13) and Australia (12), China (n=1), Brazil (1), New Zealand (3), Spain (2), UK (1), Fiji (1), Tonga (1), France (1), Sweden (1), and one study which was conducted in eight European countries (Belgium, Cyprus, Estonia, Germany, Hungary, Italy, Spain and Sweden) |
| Tallon et al., 2021^88^ | To summarize the most recent evidence to assess the impact of school-based nutrition education programs, using a technology-based approach on adolescents’ nutrition-related knowledge and behaviour | No restrictions | Adolescents aged 12-18 years | Interventions with a nutrition-related primary outcome (knowledge and/or behaviour) and include only school-based interventions that used Information and Communication Technologies | 1 month to 3 years | 13/13 | Belgium (1), UK(1) USA (4), Taiwan (1), Canada (1), Austria, Belgium, Sweden, Greece, Germany (1), Finland (1), Denmark (1), France (1), China (1) |
| Varman et al., 2021^89^ | To examine the effects of experiential learning activities among a broader age range of children (0–12 years), and in a broader range of settings including both school/pre-school and community settings, to provide a more comprehensive assessment of experiential learning opportunities for children. | RCTs or CRCTs | Children aged 0-12 years | Intervention with experiential learning activity from all settings (e.g., school, after school programs, preschools/early childhood education and care centres, farms, and school canteens) and had at least one outcome related to food or nutrition behaviour, attitudes, or knowledge. | 1 session to 2 years | 17/25 | Belgium (1), USA (16), England (1), Norway (2), Lebanon (1), Spain (2), Nepal (1), Bhutan (1) |
| Vega-Salas et al., 2023^90^ | To systematically assess the effectiveness of interventions and policies targeting the school environments for preventing/reducing overweight or obesity among school children in Latin America and the Caribbean | RCTs, NRCTs, cohort studies | Children and adolescents aged 6-17 years | Interventions, policies or regulations that change food and/or physical environment within and around the schools | 10 weeks to 3 years | 5/9 | Mexico (4), Argentina (1), Brazil (1), Chile (1), Colombia (1), Ecuador (1) |
| Verjans-Janssen et al., 2018^91^ | To study the effectiveness of primary school- PA, sedentary behaviour and nutrition interventions with direct parental involvement on children’s BMI or BMI z-score, PA, sedentary behaviour and nutrition behaviour and categorize intervention components into targeted socio-cognitive determinants and environmental types using the Environmental Research framework for weight gain prevention | RCTs, QED, pretest-post-test design, repeated cross-sectional design | Children aged 4-12 years | School-based interventions with at least one of the following approaches: (a) modifications to the physical environment, such as providing FV at school or creating activity-friendly playgrounds; (b) adjustments to the social environment, such as training school staff in health promotion or implementing teacher-led activity breaks; (c) policy changes, such as rules encouraging FV consumption or active transportation to school; and (d) economic support, such as funding for PA initiatives or the provision of FV | 10 weeks to 4 years | 22/25 | USA (9), Greece(2), Mexico(1), Norway (1), China(5), Italy(1), Australia(3), Chile(1), Germany (2) |
| von Philipsborn et al., 2019^92^ | To assess the effects of environmental interventions (excluding taxation) on the consumption of sugar-sweetened beverages and sugar-sweetened milk, diet-related anthropometric measures and health outcomes, and on any reported unintended consequences or adverse outcomes | RCT, NRCT, CBA, ITS | Any participants, including children, teenagers and adults | Interventions implemented at an environmental level, reporting effects on direct or indirect measures of sugar-sweetened beverage intake, diet-related anthropometric measures and health outcomes | 3 months to 6 years | 19/58 | USA (38), Australia (4), Netherlands (3), Canada (2), UK (2), Brazil (1), Chile (1), Germany (1), Italy (1), Mexico (1), New Zealand (1), Norway (1), Peru (1), Vietnam (1) |
| Wahi et al., 2021^93^ | To understand if, among Indigenous children, programs aimed at obesity prevention and/or the promotion of healthy lifestyle behaviours, including nutrition and PA, are effective in preventing obesity. | RCT and QED | Indigenous children aged ≤18 years | Interventions focused on preventing obesity or promoting healthy lifestyle behaviours related to nutrition and/or PA | 5 weeks to 8 years | 20/34 | Canada (15), Australia (4), USA (14), New Zealand (1) |
| Wall et al., 2022^94^ | To evaluate the effect of school-provided meals on educational outcomes in preschool and primary school children in sub-Saharan Africa | RCTs and cluster RCTs, prospective cohort studies, CBA, pre-post-test design | Children aged 3-12 years in sub-Saharan Africa | School feeding programs | 9 months to 2 years | 9/9 | Uganda (1), Senegal (1), Ethiopia (1), Kenya (2), South Africa (1), Burkina Faso (2), and Malawi (1) |
| Wang et al., 2021^95^ | To assess the impacts of school feeding programs on educational and health outcomes of children and adolescents in low- and middle-income countries | RCTs and controlled before-after studies | Children and adolescents aged 6-19 years | Provision of food or beverages in the school, including formal meals (breakfast, lunch, or dinner) or snacks | 1 week to 2 years | 39/39 | China (5), India (5), Jamaica (3), Ghana (2), Indonesia (2), Iran (2), Kenya (2), Peru (2), Philippines (2), South Africa (2), Uganda (2), Vietnam (2), Bangladesh (1), Benin (1), Burkina Faso (1), Colombia (1), Lebanon (1), Papua New Guinea (1), Senegal (1), Thailand (1) |
| Whitehead et al., 2021^96^ | To determine the effectiveness of nurse-led interventions to prevent childhood and adolescent overweight and obesity | Randomised trials | Children and adolescents aged ≤18 years | Nurse-led interventions to prevent child and adolescent overweight and obesity | 2 to 60 months | 2/18 | USA(6), Netherlands (1), Sweden (2), Iceland(1), UK (3), New Zealand (1), Australia (1), Canada (1), Finland (1), Hong Kong (1) |
| Xu et al., 2020^97^ | To describe the characteristics and identify effectiveness of school-based intervention and facilitating factors for successful intervention to promote adolescent health in low and middle income countries of WHO Western Pacific Region | RCTs and NRCTs having pre-and post-test | Adolescents aged 10-19 years | Health-related interventions in primary or secondary schools, such as health promotion, screening for health and psychosocial conditions, creating safe and nurturing learning environments. | 6 weeks to 36 months | 1/8 | China (4), the Philippines (1), Mongolia (1), Cambodia (1), Malaysia (1) |
| Zuair et al., 2022^98^ | To conduct a systematic and meta-analytic review of the effects of media health literacy interventions in school settings on adolescents’ body image, that included body image concerns, eating concerns, and thin-internalization attitudes | Experimental study design | Adolescents aged 10-18 years | Media literacy or media health literacy interventions in school settings | Total duration of studies not reported | 16/16 | Australia (7), USA (3), Canada (1), Israel (1), Spain (4) |

**Abbreviations:** RCT; NRCT: Non-randomized controlled trials; CBA controlled before-after studies PA: Physical activity; HE: Healthy eating

**Table S2.** Tools reported in the assessment of other dietary intake outcomes in school-based nutrition interventions

| **Name of Tool** | **Methods** | | **Validation assessment** |
| --- | --- | --- | --- |
|  | **Outcome**  **Tool type (time frame)** | **Administration (Construction and Collection)** |  |
| **Subjective measures** | | | |
| **Child and Diet Evaluation Tool (CADET)^99,100^** ([www.nutritools.org](http://www.nutritools.org)) | DI (Overall + FV)  24HR (1 day) | The 115-item paper-based food checklist assesses all aspects of the diet, with a focus on FV consumption. It prospectively records all foods consumed over one 24-hour period, including a retrospective breakfast section. The tool has three parts: the first part is completed by a parent or caregiver, the second part by a trained fieldworker, dinner supervisor or parent classroom assistant, and the third part is self-completed by the child with assistance from the classroom teacher. | Yes, in 3-7^99^ and 8-11^100^ years olds in England |
| **Day in the Life Questionnaire (DILQ)^101^** ([www.nutritools.org](http://www.nutritools.org)) | DI (FV)  24HR (1 day) | The 17-item paper-based classroom exercise assesses the number of servings of FV consumed over the past 24 hours. The self-reported tool uses words and pictures to help the child recall and describe various activities from the previous day, including their food intake. | Yes, in 7-9 years olds in England^101^ |
| **Block Food Screener for Ages 2–17 years 2007^102^**  ([www.nutritionquest.com](http://www.nutritionquest.com)) | DI  FFQ (1 day or 7 days) | The tool, available in paper and electronic formats, assesses children's intake by servings per food group. It features two versions: the ‘Block Kids 2-17 Screener – Yesterday’ (for food consumed the previous day) and the ‘Block Kids 2-17 Screener - Last Week’ (for food consumed over the past week). It is self-reported by children with parental or caregiver assistance as needed. | Yes, in 10-17 years olds in the USA^102^ |
| **Youth/Adolescent Questionnaire (YAQ)^103,104^** | DI  FFQ | The 152-item self-administered questionnaire assesses dietary patterns in children and adolescents over the past year. It is based on the validated adult Nurses’ Health Study FFQ which was modified to include 27 snack items as a separate category and several other foods. Respondents report how frequently they consumed specific foods (e.g., yogurt, potato chips, and noodles) over the past year, with response categories varying by food type. Popular items (e.g., apple juice) have options for days, weeks and months, while less popular items (e.g., raisins) have weekly or monthly options. The frequencies for each item are synthesized and transformed into a series of nutrient indices representing daily intake. | Yes, in 9-19 years olds in the USA^104^ |
| **Goals for Health Questionnaire**^105,106^ | DI (fat, FV, fibre)  FFQ | The self-administered tool, developed as part of a school-based cancer prevention program, and was refined based on the Youth/Adolescent Questionnaire (YAQ). It consists of 25 food frequency items and 10 supplemental questions that assess fat, FV and fibre intake. | Yes in sixth- and seventh-grade students in the USA^105^ |
| **Australian Child and Adolescent Eating Survey (ACAES)^107^** | DI, SB  FFQ (6 months) | The self-administered tool assesses habitual intake of 120 food and beverage items over the past 6 months, with 15 additional questions on food behaviours and sedentary behaviour. It was developed based on the Youth/Adolescent Questionnaire (YAQ). | Yes, in 9-18 olds in Australia^107^ |
| **Pizza Please^108^** | DI + Nutrition knowledge  Evaluation tool | The tool assesses dietary behaviour and nutrition knowledge in primary school-aged children through an interactive game and questionnaire. The game includes a life-size pizza with detachable toppings and place mats where players earn toppings by correctly answering mealtime questions. The questionnaire consists of 24 questions on dietary behaviour and 16 questions on nutrition knowledge related to primary school-aged children. Dietary behaviour is evaluated using "yes/no" questions about meal and snack frequency, while nutrition knowledge is assessed with matching and item-elimination questions on food groups, nutrient associations, and dietary habits. | Not found |
| **Modified version of Behavioural Risk Factor Surveillance System Questionnaire (BRFSS)^109^**  (<https://www.cdc.gov/brfss/>) | FV  Survey | The self-report telephone survey was developed by CDC to collect data on preventive health practices and risk behaviours linked to chronic diseases, injuries and preventable infectious diseases. The modified version assesses usual FV intake. The instrument includes six items that measure the frequency of consumption of fruit, fruit juices, green salad, potatoes (excluding French fries, fried potatoes, and potato chips), carrots and vegetables over the past year. These items were weighted and summed to estimate the average daily FV servings. | Not found |
| **24-hour diet recall** | DI  24HR (1 day) | Several systematic reviews reported that the studies they included used 24HR; however information on the name of 24HR used was not available from systematic reviews. | Not reported |
| **Food Frequency Questionnaire** | DI  FFQ | Several systematic reviews reported that the studies they included used FFQ; however information on the name of FFQ used was not available from systematic reviews. | Not reported |
| **Food diary** | DI  Food diary | Several systematic reviews reported that the studies they included used food diary or record; however information on the name of FFQ used was not available from systematic reviews. The length of food diaries varied, including 3 to 7 days. | Not reported |
| **Mediterranean Diet Quality Index for children and adolescents (KIDMED index)**^110^ | MD adherence  Index | The index comprises a 16-question test, either self-reported or conducted through an interview (e.g., by a paediatrician or dietitian), to assess MD adherence. Questions are answered with "yes" or "no", with negative aspects scored as -1 and positive aspects as +1. The total score (i.e., sum of all responses) ranges from 0 to 12 points and is categorized into three levels: ≥8 = ’optimal MD’, 4–7= ‘average MD adherence, with room for improvement towards MD patterns’ and ≤3= ‘very low diet quality.’ | Yes, in 12.5-17.5 years olds in European countries^111^ |
| **Revised children’s diet quality index (RCDQI)^112^** | Diet quality  Index | The 13-component index assesses children’s diet quality based on adherence to the dietary recommendations. Adolescents who meet the recommended consumption levels receive full points, from 2.5 to 10 per component. Points are reduced proportionally for suboptimal intakes or overconsumption. The total possible score was equal to 90. | Not found |
| **Meal Index of Dietary Quality (Meal IQ)**^113-115^ | Diet quality Index | The tool assesses the overall dietary quality of lunches for children aged 7–13 years based on seven components: total fat, saturated fat, whole grains, snack products, fruit, vegetables and fish. The total score ranges from 0 to 28, with each of component scored from 0 (non-compliance) to 4 (full compliance), indicating adherence to dietary recommendations. | Yes, in 7–13 years olds in Denmark^114^ |
| **Ernährungsmusterindex^116^** | Healthy nutrition  Index | The index measures changes in dietary behaviour through items indicating healthy and unhealthy food choices, such as FV (cooked, raw, frozen, and tinned), whole-grain bread, soft drinks, fast food, chocolate and snacks (e.g., crisps or pretzel sticks). The questions were derived from the German National Health Interview and Examination Survey for Children and Adolescents (KiGGS). The index is completed by both children and their parents. | Not found |
| **Eating Behavior Questionnaire (EBQ)^117^** | DI  Questionnaire | The tool assesses the children’s main meal and snacking behaviour, including the frequency of consumptions of breakfast, lunch and dinner, as well as morning tea, evening tea and supper. Children report the number of days these meals and snacks are consumed in a week. | Not found |
| **Food Habits Questionnaire (FHQ)**^118,119^ | DI (Overall)  FFQ (past month) | A 21-item series of questions about frequency of consumption of a variety of foods, including high and low-fat food items (e.g., FV, tacos and other fast foods). Students responded on a ﬁve-option response format to indicate how often they consumed the foods over the previous month, ranging from never to always. Low-fat items are reverse scored, and an algorithm was used to calculate percentage of fat, such that the resulting total score indicates percentage of fat in food consumed during the previous month. | Not found |
| **National Cancer Institute Fruit and Vegetable Screener**^120,121^ | DI (FV)  Screener (past month) | The 19-item screener assesses the usual frequency of consumption of 10 categories of FV over the past month. It estimates daily servings based on the 1992 USDA [Food Guide Pyramid](https://www.sciencedirect.com/topics/medicine-and-dentistry/food-guide-pyramid)’s defined servings. | Not found |
| **Beverage Questionnaire-10 (BEVQ-10)**^121,122^ | DI (beverage)  FFQ (past month) | The FFQ estimates habitual mean daily intake of beverages, including sweetened juice, soft drinks (regular and diet), sweetened tea, energy drinks, 100% fruit juice and milk (whole, 2%, skim) (grams and kcals consumed). Respondents are asked to indicate “how often” and “how much” of a beverage they consumed in the past month. Coding was based on the standard protocol developed and validated by Hedricks et al (2012). | Yes^122^ |
| **Energy Balance Related Behavior Questionnaire (EBRB)**  (Shrewsbury et al, 2020) | DI, PA, SB  Questionnaire | The EBRB items were adapted from NSW SPANS (see above) and explored several behaviours: frequency of eating breakfast, daily intake of FV, daily intake of SSB, frequency of achieving 60 minutes of moderate-to-vigorous physical activity per day, and screen time during free time on school days. Intentions to change these behaviours in the following month were also assessed, excluding SSB consumption. The post-program questionnaire included additional questions about students' perceptions of the intervention program, such as program recommendation to peers, key program messages learned and discussions with family about the program. | Not found |
| **Technological tools** | | | |
| **Web-based Dietary Assessment Software for Children (WebDASC)^123^** | DI  Food record (7 days) | The online interactive food record, developed for the OPUS School Meal Study, assesses and monitors children’s dietary habits. It is self-administered by children, with or without parental support, over seven consecutive evenings. An animated armadillo guides children through six daily eating occasions (breakfast, morning snack, lunch, afternoon snack, dinner, and evening snack) and encourages them to record foods and beverages consumed. It uses a database of 1300 food items from the Danish National Survey of Diet and Physical Activity. A type-in format is available for foods not listed through category browsing or free-text search. Portion sizes are estimated using four digital images to match the closest portion size. | Yes, in 8-11 year olds in Denmark^123^ |
| **Diet-A Mobile App^124^** | Dietary self-monitoring Mobile App | The app allows users to record food intake via voice or text input. Nutrient intake is calculated using databases from the Korea Ministry of Food and Drug Safety, National Rural Living Science Institute and the Korea National Health and Nutrition Examination Survey. It provides personalized real-time feedback on energy, carbohydrates, protein, fat, sodium, calcium and iron intakes, comparing these to the Dietary Reference Intakes for Koreans 2010. The app aims to prevent obesity, diabetes, hypertension and dyslipidaemia by alerting users if their nutrient intake exceeds recommended levels over the past week. It also reminds users to log meals at specific times and suggests healthier food options. | Not found |
| **Cloud Diet Assessment System (CDAS)^125^** | Dietary self-monitoring | The system is a novel strategy developed by study authors in Taiwan to provide automatic feedback on the nutritional intake of learners through a meal analysis algorithm. | Not found |
| **Objective measures** | | | |
| **Quarter waste (plate or tray)**   - **Digital Photography Method** - **Visual Observation Method** | Selection, DI, Waste  Quarter waste | Quarter waste with the digital photography or visual observation methods has been reported in several systematic reviews for the assessment of school meals or designated food items (e.g., FV and milk) selection, consumption, and waste. Some reviews detailed the duration of the measurement period (e.g., across three lunch periods), validation status, and indicated that the measurements were conducted by trained observers following a standardized protocol. For example, Comstock scale (Comstock et al., 1981) was cited as used in the examination of food remnants left by children across lunch periods (Blom-Hoffman et al., 2004; Jones et al., 2015). This scale has 6 points. | Not reported |
| **Direct Weighing** | DI, Waste | Direct weighing of food on plate/tray before and/or after cafeteria service was mentioned in several systematic review for the assessment of consumption and waste of school meal components. Some mentioned that the food was weighed by trained observers following a standardized protocol. | Not reported |
| **Food insecurity, hunger and satiety measures** | | | |
| **Teddy the Bear hunger and satiety scale**^126^ | Hunger and satiety  Scale | The scale features five black-and-white cartoon bear silhouettes with varying sizes of ovals in their stomach area. The size of the oval increases proportionally with the amount of food consumed and consequently the satiety level of the bear. Each silhouette is accompanied by descriptive vignettes that explain the bear’s level of hunger and satiety on a 5-point Likert scale, ranging from 1 (very hungry/not satiated at all) to 5 (very full/not hungry at all). Children hear a story about Teddy the Bear, rate the bear’s hunger at two points in the story and also rate their currently perceived hunger/satiety state. | Yes, in 5-9 years olds in England^126^ |
| **8-item hunger/food insufficiency questionnaire**^127,128^ | Hunger | The 8-item, parental-completed hunger/food insufficiency questionnaire was developed by the Community Childhood Hunger Identification Project (CCHIP). Children were classified as ‘hungry’ if the parent responded positively to 5 or more of the 8 questions concerning hunger in the past year. Children were classified as ‘at risk for hunger’ if the parent responded positively to one or as many as 4 of the 8 food insufficiency questions. If the parent did not respond positively to any of the 8 food insufficiency questions, the household and child were classified as ‘not hungry’. | Not found |
| **5-item version of the Child Hunger Index Child Report survey**^127^ | Hunger | The 5-item version of the CCHIP survey, the Child Hunger Index Child Report (CHI-C) were administered to children. Children who responded positively to 2 or more of the CHI-C questions were classified as ‘hungry’. When the child responded positively to one of the questions, the child was classified as ‘at risk for hunger’ on the CHI-C. Those who did not respond positively to any of the items were classified as ‘not hungry’. | Yes in 6-12 years olds in the USA^129^ |
| **Food Security Survey Module (FSSM)**^130,131^ | Food insecurity  Questionnaire | The questionnaire includes 18 questions that measures reactions to food insecurity, such as stress from lack of food and inadequate food quality and quantity and to determine the level of food insecurity. Questionnaires are completed by parents. Responses are summed up to produce a score ranging from 0 to 18, with higher scores indicating greater food insecurity. Scores categorize households into four levels: 'food security' (score 0–2), 'food insecurity without hunger' (score 3–7), 'food insecurity with moderate hunger' (score 8–12), and 'food insecurity with severe hunger' (score 13–18). | Not found |

**Abbreviations:** **DI** dietary intake; **PA** Physical activity; **SB** sedentary behaviour; **FV** fruit and vegetables; **SSB** sugar sweetened beverages; **MD** Mediterranean diet; **FFQ** food frequency questionnaire; **24HR** 24-hour dietary recall; **CDC** Centres for Disease Control and Prevention

**Table S3.** Tools reported in the assessment of diet-related attitudinal and cognitive outcomes in school-based nutrition interventions

| **Name of Tool** | **Outcomes** | **Methods/Administration** | **Validation** |
| --- | --- | --- | --- |
| **Food Knowledge Questionnaire^132^** | Knowledge | The questionnaire was developed by an interdisciplinary research team, including a nutritionist, a psychologist and a computer scientist. It includes 49 healthy and 41 unhealthy food items, selected based on interviews with children prior to the study. Participants categorize each food as "healthy", "unhealthy" or "I do not know" following the UK Food Standard Agency’s traffic light colour coding for nutrient content. The total score is the sum of correctly answered questions. | Not found |
| **Dairy Self-Efficacy Scale (DSES)^133^** | Knowledge and confidence (Dairy) | The scale assesses adolescents’ knowledge and confidence for behaviour change related to dairy food consumption along with demographic and food frequency information. The self-efficacy section includes 10 knowledge items and 15 confidence questions, rated on a 5-point Likert scale from strongly disagree to strongly agree. he food frequency section, adapted from the Youth Risk Behavior Survey (CDC, 2005), includes seven questions scored from 1 to 7, where higher scores indicate more frequent dairy consumption. | Not found |
| **Food Choices Scale for Children^134^** | Food neophobia | The scale assesses knowledge, attitude, perception and willingness to try new foods. It was adapted from the validated Food Neophobia Scale and the Food Neophobia Scale for Children. The scores were cumulated into a single score. | Not found |
| **Nutrition Behavior Inventory^135^** | Nutrition knowledge, attitude and behaviour | The inventory includes 16 items for perceived nutritional knowledge, 12 items for perceived nutritional attitude and 9 items for perceived nutritional behaviour in the original version. The nutritional knowledge scale has three options, "false" (1), "I do not know" (2) and "correct" (3), where higher score indicates more awareness of the proper nutritional behaviours. Perceived nutritional behaviour ranges from 1 (I disagree) to 5 (I totally disagree), where higher score indicates positive attitudes toward proper nutritional behaviours. Perceived nutritional behaviour ranges from 1 to 4, where higher score is related to the most correct and highest score. | Not found |
| **Questionnaire used to evaluate effectiveness of school-based nutrition and food safety education intervention^136^** | Nutrition knowledge, habits, attitude, behaviour | The questionnaire was designed by study authors based on literature on nutrition and food safety knowledge questionnaire in primary and high school students. The questionnaire includes four sections: basic information; nutrition knowledge; eating habits, attitude, and behaviour; and food safety knowledge. | Not found |
| **Beverage Frequency Questionnaire (BFQ)^137^** | Beverage knowledge, attitude and behaviour | The self-report questionnaire includes 19 questions on weekly consumption of specific beverages, 4 questions on beverage intake habits at school, 6 questions on attitudes towards beverages, 11 questions on knowledge of healthy foods and beverages and 3 demographic questions. Attitude and knowledge questions were answered using the options: agree, somewhat agree and disagree. Beverage intake questions are rated on a scale from more than once per day to seldom or never. Scores for each knowledge and attitude response are calculated based on the provided answers. Knowledge and attitude scores were calculated separately and then totalled. | Not found |
| **Food Pairing Questionnaire^138^** | Nutrition knowledge, preference and behaviour | The questionnaire includes three sections: knowledge, preference and behaviour. In each section there were 18 pairs of pictures of foodstuffs, one signifying the healthy choice and the other the unhealthy one. Each section contained the same 18 food pairs, and the respondents were asked to circle which food they ate most often, which they liked best and which food they thought was better for them. This type of visual instrument was felt to be appropriate for the age group. As it had been validated on a similar group of children in the United States no changes were made apart from altering the names of some foodstuffs to colloquial English. | Yes^138^ |
| **Knowledge, Self-Efficacy and Intentions Questionnaire (KSIQ)**^139,140^ | Knowledge, self-Efficacy and intentions | The questionnaire was adapted from the Pro Children questionnaire which was initially designed to assess the determinants of FV consumption in 10-11-year-old European children. It included questions on self-rated intake, knowledge, attitudes, liking, subjective norm, active parental encouragement, general self-efficacy, intention, habit, preferences, availability and perceived barriers. Although the questionnaire has not been validated in an Aboriginal population, a formative evaluation with the snack program coordinator ensured its appropriateness and confirmed its face validity for students in Fort Albany FN. The KSIQ was administered in-class to all grade six to eight students, both before and after the education program. | Yes^139,140^ |
| **Waterloo Web-based Eating Behavior Questionnaire (WEB-Q)^139,141^** | 24HR  1 day  food and PA behaviours | The web-based survey assesses food-related knowledge, attitudes, intentions and behaviours among children and adolescents. It includes a 24-hour dietary recall, a FFQ and questions where students report their food intake from the previous day, using prompts, pictures and comparisons for portion size estimation. The tool has been extensively used to gather nutrition and physical activity data from both non-Aboriginal and First Nation students in Canada. | Yes, in students aged sixth to 10th grade from Ontario and Alberta and from First Nations students from Ontario**^139,141^** |
| Gavaravarapu et al., 2016 ^142^ | Nutrition label use and understanding | The study evaluated practices related to the use of nutrition labels with 5 self-reported questions (e.g., “Do you read the sugar content when buying chocolate?”) and knowledge of food label information with 1 question, “Is nutrition information present on this label?”. | Not found |
| **Garden Vegetable frequency questionnaire (GVFQ)^143^** | Vegetables knowledge, attitudes and behaviour | The questionnaire is a paper-based self-administered tool designed to measure the consumption and preferences for vegetables commonly grown in school gardens. It features pictures and names of 22 vegetables, with space for students to add any additional vegetables they may have consumed. The questionnaire assesses types of vegetables consumed the previous day, their frequency of consumption and preferences. Students typically complete the GVFQ in a classroom setting. | Not found |
| **Food label literacy^144^** | Nutrition label understanding | The quiz assesses the ability to distinguish between more and less healthful foods using a validated test instrument with Nutrition Facts panels and ingredient lists. | Not found |

**Table S4.** Tools reported in the measurement of social and emotional well-being and behaviour domain in school-based nutrition interventions

| **Name of Tool** | **Outcome** | **Description** | **Validation** |
| --- | --- | --- | --- |
| **Knowledge and behaviour related to health and lifestyle** | | | |
| **Dutch Health Behavior in School-aged Children (HBSC) questionnaire^145,146^** | Health behaviours | The self-report survey covers a range of health and wellbeing topics, including demographic factors (e.g., age and state of maturation); social background (e.g., family structure and socio-economic status); social context (e.g., family, peer culture, school environment); health outcomes (e.g., self-rated health, injuries, overweight and obesity); health behaviours (e.g., nutritional habits, PA and weight reduction behaviour); and risk behaviours (e.g., smoking, alcohol use, cannabis use, sexual behaviour, bullying). | Not found |
| **Health Behavior Questionnaire**^147,148^ | Health behaviours and attitudes | The questionnaire measures health behaviours and attitudes in children, including items on the following scales: Usual Food Choices (14 items), Dietary Self-Efficacy (15 items, e.g., “How sure are you that you can eat a baked potato instead of French fries?”), and PA Self Efficacy (5 items, e.g., “How sure are you that you can choose to jog during recess?”) scales. | Not foundcheck |
| **Youth Risk Behavior Survey (YRBS)^149^** | Health behaviours | The survey is part of the Youth Risk Behavior Surveillance System developed by the CDC to monitor health risk behaviours that contribute to mortality, morbidity, disability and social problems among youth. These include behaviours that result in unintentional and violence; tobacco use; alcohol and other drug use; sexual behaviours that result in unintended pregnancies and sexually-transmitted diseases, including HIV infection; dietary behaviours; and physical activity. | Not found |
| **Adolescent Behaviours, Attitudes and Knowledge Questionnaire (ABAKQ)^150^** | Health knowledge, attitudes and behaviours | The self-report questionnaire includes sections on demographics, health behaviours and mental wellbeing. It focuses on key behaviours, such as nutrition, physical activity, sedentary behaviours, perceptions of school environment (teachers, canteens, opportunities for physical activity/healthy nutrition), home environment (role of parents/siblings), and neighbourhood environment. The mental wellbeing subsection assesses health related quality of life and depressive symptomatology. | Not found |
| **Healthy Habits Survey^151^** | Health behaviours | The 10-question survey, completed by caregivers, assesses the health habits of children aged 2 to 9 years. It covers physical activity, screen time, fast-food intake, family meal frequency, FV intake, SSB intake and the presence of a television or computer in the bedroom. The survey was modified to replace free text answers with multiple choice responses and to add a question on children’s sleep duration at night. | Not found |
| **Social and emotional wellbeing** | | | |
| **Mindful Attention Awareness Scale (MAAS)^152^** | Mindful Awareness | The 15-item scale assesses trait mindfulness or awareness of present experience, with items like “I find myself preoccupied with the future or the past” and “I find myself doing things without paying attention”. Responses are given on a 6-point Likert scale from 1 (almost always) to 6 (almost never), where higher scores indicate higher degree of mindfulness trait. | Yes^153^ |
| **Children’s Depression Inventory (CDI)^154,155^** | Depressive symptomatology | The 27-item self-report scale measures depressive symptoms in children and adolescents. Total scores range from 0 to 54, where higher scores indicate greater symptomatology. A score of 13 is recommended as a threshold for detecting depressive disorders. | Yes |
| **Mood and Feelings Questionnaire (MFQ)^156,157^** | Depressive symptomatology | The screening tool assesses depressive symptoms in children and young people aged 6 to 19. It has six versions: child self-report, parent-report and adult self-report, each available in a long (33 questions) and short version (13 questions). The items includes descriptive phrases related to mood states, and respondents are asked to indicate how they felt or acted in the past two weeks, rated on a 3‐point scale (0 = not true, 1= sometimes and 2 = true). A total score is obtained by summing the point values of responses for each item, with higher scores indicating more severe depressive symptoms. | Yes |
| **Strengths and Difficulties Questionnaire (SDQ)^158^**  ([www.sdqinfo.com](http://www.sdqinfo.com)) | Emotional and behavioural screening | The emotional and behavioural screening tool is designed for children and young people aged 2-17 years for use in research, clinical or educational settings. The SDQ consists of 25 items across 5 scales: Emotional Problems, Conduct Problems, Hyperactivity, Peer Problems and ProSocial. Each item describes a positive or negative trait and is rated on 3-point Likert scale (0 = ‘Not True’, 1 = ‘Somewhat True’, 2 = ‘Certainly True’). A Total Difficulties score can be obtained by summing the scores of all scales except the Prosocial scale. | Yes |
| **Paediatric Quality of Life Inventory (PedsQL)**^159^ ([www.pedsql.org](http://www.pedsql.org)) | Health-related quality of life | The modular instrument measures health-related quality of life in children and adolescents aged 2–18 years. It includes four scales: Physical functioning (8 items), Emotional functioning (5 items), Social functioning (5 items) and School functioning (5-items). Both children (self-report) and their parents (proxy report) rate their perceived quality of life on a 5-point Likert scale (0 = never a problem to 4 = almost always a problem). Higher scores indicate higher health-related quality of life. Summary scores can be calculated for Physical Health (8 items), Psychosocial Health (15 items), and Total Scale (23 items). | Yes |
| **KIDSCREEN^160^** | Health-related quality of life | The questionnaire assesses subjective health and the psychological, mental and social well-being in children and adolescents. It covers aspects, such as PA and energy levels, emotions, depressive moods, stress, ability to enjoy recreational activities, socialising, relationships with parents/carers and peers and perception of cognitive capacity and school performance. Each item is rated on a 5-point scale. It is available in three versions: KIDSCREEN-52, KIDSCREEN-27 and KIDSCREEN-10, and has been adapted into multiple languages. | Yes |
| **Eating disorders** | | | |
| **Dutch Eating Behavior Questionnaire (DEBQ)^161^** | Restrained, emotional and external eating | The 33-item questionnaire assesses eating behaviours associated with the development of overweight in adults through three scales: emotional eating (13 items), external eating (10 items) and restrained eating (10 items). Responses are rated on a 5-point Likert scale from 1 (never) to 5 (very often), where higher scores indicate greater endorsement of the behaviour. The questionnaire is available in multiple languages and has adaptations for children (self-report or parental report) and older populations. | Yes |
| **Eating Attitudes Test (EAT)^162,163^** | Eating disorder symptoms | The self-report measure, available as 40-item (EAT-40) and (EAT-26) 26-item versions assesses attitudes and behaviours related to eating disorders. Items are rated on a 6-point Likert scale, from 1 (always) to 6 (never), based on the frequency of behaviours. The EAT-26 yields a “referral index” based on three criteria: 1) the total score from answers to the EAT-26 questions; 2) answers to the behavioural questions related to eating symptoms and weight loss, and 3) the individual’s body mass index calculated from their height and weight. Scores above 20 typically indicates disordered eating behaviour. | Yes |
| **Children’s Eating Attitude Test (ChEAT)^164^** | Eating attitudes and Disordered eating | The 26-item self-administered questionnaire assesses eating attitudes and behaviours in children aged 8-13 years. It is a simplified version of the Eating Attitudes Test (EAT-26) used for adults. The items are rated on a 6-point Likert scale from ‘never’ to ‘always’. A total score is derived by summing the points for each item. Higher scores indicate more problematic eating attitudes and behaviours, with scores of 20 or above generally considered significant. The test has been adapted in several countries. | Yes |
| **Eating Disorder Inventory (EDI)^165-167^** | Eating disorders | The self-report measure assesses psychological and behavioural traits common in anorexia nervosa and bulimia. The original version includes 64 questions across eight subscales: Drive for thinness, Bulimia, Body dissatisfaction, Ineffectiveness, Perfectionism, Interpersonal distrust, Interoceptive awareness and Maturity fears. Each question is answered on a 6-point scale from "always" to "never". Subscales scores can be used separately or aggregated to form a total score. There are two subsequent revisions: the Eating Disorder Inventory-2 (EDI-2) and the Eating Disorder Inventory-3 (EDI-3). | Yes^165,167^ |
| **Kids Eating Disorder Survey (KEDS)^168^** | Figure/body dissatisfaction | The 14-item self-report instrument assesses eating disorders pathology in children by identifying predictors of weight dissatisfaction and purging behaviour. The scale includes questions adapted from the Eating Symptoms Inventory along with 8 child figure drawings. | Not found |
| **Body Esteem Scale for Adolescents and Adults (BES)^169^** | Body esteem | The 30-item scale assesses affective self-evaluations of body or appearance in adolescents and adults through three subscales: Appearance (general feelings about appearance), Weight (weight satisfaction), and Attribution (evaluations attributed to others about one's body and appearance). Items, for example "I like what I look like in pictures," "I am satisfied with my weight" and "My looks help me get dates," are rated on a 5-point scale (0 = Never to 4 = Always), where higher scores indicate higher body esteem. | Yes**^169^** |
| **Body Esteem Scale^170^** | Muscle esteem | The 4-item scale measures muscle esteem in children with statements: “I really like my muscles”, “I’m proud of my muscles”, “I think I have good muscles” and “I wish I had more muscles”. Scores range from 0 to 4, where higher scores indicate higher muscle satisfaction (the fourth item was reverse scored). | Not found |
| **Body Change Inventory**^170,171^ | Body change strategies | The 4-item tool assesses children’s use of body change strategies, including diet and/or exercise to increase body size and muscle size, as well strategies to decrease body size. Children are asked to indicate the frequency of the behaviour on a 5-point Likert scale from “never” (1) to “always” (5). Scores range from 4 to 20, where higher scores indicate more frequent use of body change strategies. | Yes^170,171^ |
| **Sociocultural Influences on Body Image and Body Change Questionnaire^170,172^** | Influences on body image | The tool assesses the influence of peers, parents and the media on children’s body image through three items per subscale (Peers, Parents and Media). The items assess pressure related to weight loss, fitness and muscle gain on a 5-point Likert scale from “never” (1) to “always” (5). Each subscale scores range from 3 to 15, where higher scores represent higher perceived pressure to lose weight, become fitter or gain muscle. | Not found |
| **Male Physical Attributes Investment Scale^170,173^** | Male physical attributes | The 8-item scale assesses attitudes towards physical attributes in boys aged 11–15 years. Statements about the importance of boys' athleticism and physical superiority (e.g., "It is important for guys to be able to physically defend themselves") are rated on a 5-point scale (from “strongly disagree” (1) to “strongly agree” (5)). Scores are summed to create a total score ranging from 7 to 35, where higher scores indicate stronger endorsement of masculine gender ideals. | Not found |

**Table S5.** Tools reported in the assessment of education outcomes

| **Name of Tools** | **Outcome** | **Measure** | **Validation** |
| --- | --- | --- | --- |
| **Raven’s Coloured Progressive Matrices test** ([www.pearsonassessments.com](http://www.pearsonassessments.com)) | Intelligence | The non-verbal test assesses abstract reasoning/non-verbal ability used widely across different age groups. It comprises 60 [multiple choice](https://en.wikipedia.org/wiki/Multiple_choice) questions, listed in order of increasing difficulty | Not found |
| **Cambridge Neurological Test Automated Battery (CANTAB)^174^**  ([www.cambridgecognition.com](http://www.cambridgecognition.com)) | Cognitive function | The computer-based cognitive assessment system uses 25 neuropsychological tests, administered via a colour touch-sensitive screen computer. The tests assess cognitive functions, such as general memory and learning, working memory and executive function, visual memory, attention and reaction time, semantic/verbal memory, decision making and response control. The system is used across several age groups and patients with psychiatric and neurological conditions. | Yes  ([www.cambridgecognition.com](http://www.cambridgecognition.com)) |
| **Weschler Intelligence Scale for Children (WISC)^175^**  ([www.pearsonassessments.com](http://www.pearsonassessments.com)) | Intelligence | The intelligence test assesses cognitive abilities and intelligence in children aged 6–16 years. The latest WISC-V edition generates five composite scores: Verbal Comprehension, Visual Spatial Index, Fluid Reasoning Index, Working Memory Index and Processing Speed Index. A Full-Scale Intelligence Quotient (FSIQ) is also generated based on seven subtests: Similarities, Vocabulary, Block Design, Matrix Reasoning, Figure Weights, Digit Span and Coding. | Yes^175^ |
| **Peabody picture vocabulary test (PPVT)^176^**  ([www.pearsonassessments.com](http://www.pearsonassessments.com)) | Receptive vocabulary | The untimed test assesses receptive vocabulary (i.e., the collection of words an individual can understand through spoken and written language) and screens for verbal ability. During the test, respondents are shown a word along with four pictures and must select the picture that best describes the meaning of the word. The test contains 228 items, divided into 19 sets of 12 items each. | Yes^176^ |
| **Cognitive tests** | Cognitive function | The effects of school-based interventions on multiple cognitive functions were measured using different tests. The details on the type of tests used were not available from systematic reviews. Cognitive functions measured include attention (e.g., forward digit span, visual search), memory (e.g., backward digit span, pattern recognition, immediate or delayed picture recognition), verbal fluency, information processing, visual perceptual organization and visual-motor coordination (e.g., coding test), continuous performance task, psychomotor speed (e.g., finger tapping), cognitive performance (e.g., addition, multiplication, number checking, logic, creativity). | Not reported |
| **Test Scores** | Academic achievement | The effects of school-based interventions on academic achievement were measured using test scores in subjects such as literacy, reading, spelling, vocabulary, maths, arithmetic, or as aggregate or end-of-term grades. Some studies indicated that the test scores were nation or statewide standardized. | Not reported |
| **Curriculum Enjoyment Scale^177^** | Curriculum enjoyment | The enjoyment of the curriculum delivered as part of the intervention was measured using four questions: ‘How did you like the home economics fruit and vegetable teaching?’, ‘Did you like the fruit and vegetable dishes you made at school?’, ‘Have you increased your skills in making fruit and vegetable dishes during this school year?’ and ‘Has the home economy teaching made you more positive towards fruit and vegetables?’. Responses were rated on a Likert-type scale from 5 to 8 and categorized into tertiles: low (2 to 5), medium (3 and 4) and high (5 to 8) levels of enjoyment. | Not found |

**Table S6.** PRISMA 2020 Checklist

| **Section and Topic** | **Item #** | **Checklist item** | **Location where item is reported** |
| --- | --- | --- | --- |
| **TITLE** | | |  |
| Title | 1 | Identify the report as a systematic review. | Page 1 |
| **ABSTRACT** | | |  |
| Abstract | 2 | See the PRISMA 2020 for Abstracts checklist. | Page 1-2 |
| **INTRODUCTION** | | |  |
| Rationale | 3 | Describe the rationale for the review in the context of existing knowledge. | Page 3-4 |
| Objectives | 4 | Provide an explicit statement of the objective(s) or question(s) the review addresses. | Page 4 |
| **METHODS** | | |  |
| Eligibility criteria | 5 | Specify the inclusion and exclusion criteria for the review and how studies were grouped for the syntheses. | Page 5 |
| Information sources | 6 | Specify all databases, registers, websites, organisations, reference lists and other sources searched or consulted to identify studies. Specify the date when each source was last searched or consulted. | Page 5-6 |
| Search strategy | 7 | Present the full search strategies for all databases, registers and websites, including any filters and limits used. | Page 6 |
| Selection process | 8 | Specify the methods used to decide whether a study met the inclusion criteria of the review, including how many reviewers screened each record and each report retrieved, whether they worked independently, and if applicable, details of automation tools used in the process. | Page 6 |
| Data collection process | 9 | Specify the methods used to collect data from reports, including how many reviewers collected data from each report, whether they worked independently, any processes for obtaining or confirming data from study investigators, and if applicable, details of automation tools used in the process. | Page 6-7 |
| Data items | 10a | List and define all outcomes for which data were sought. Specify whether all results that were compatible with each outcome domain in each study were sought (e.g. for all measures, time points, analyses), and if not, the methods used to decide which results to collect. | Page 6-7 |
|  | 10b | List and define all other variables for which data were sought (e.g. participant and intervention characteristics, funding sources). Describe any assumptions made about any missing or unclear information. | Page 6-7 |
| Study risk of bias assessment | 11 | Specify the methods used to assess risk of bias in the included studies, including details of the tool(s) used, how many reviewers assessed each study and whether they worked independently, and if applicable, details of automation tools used in the process. | Page 7-8 |
| Effect measures | 12 | Specify for each outcome the effect measure(s) (e.g. risk ratio, mean difference) used in the synthesis or presentation of results. | N/A |
| Synthesis methods | 13a | Describe the processes used to decide which studies were eligible for each synthesis (e.g. tabulating the study intervention characteristics and comparing against the planned groups for each synthesis (item #5)). | Page 6-7 |
|  | 13b | Describe any methods required to prepare the data for presentation or synthesis, such as handling of missing summary statistics, or data conversions. | Page 7-8 |
|  | 13c | Describe any methods used to tabulate or visually display results of individual studies and syntheses. | Page 7-8 |
|  | 13d | Describe any methods used to synthesize results and provide a rationale for the choice(s). If meta-analysis was performed, describe the model(s), method(s) to identify the presence and extent of statistical heterogeneity, and software package(s) used. | Page 7-8 |
|  | 13e | Describe any methods used to explore possible causes of heterogeneity among study results (e.g. subgroup analysis, meta-regression). | N/A |
|  | 13f | Describe any sensitivity analyses conducted to assess robustness of the synthesized results. | N/A |
| Reporting bias assessment | 14 | Describe any methods used to assess risk of bias due to missing results in a synthesis (arising from reporting biases). | N/A |
| Certainty assessment | 15 | Describe any methods used to assess certainty (or confidence) in the body of evidence for an outcome. | N/A |
| **RESULTS** | | |  |
| Study selection | 16a | Describe the results of the search and selection process, from the number of records identified in the search to the number of studies included in the review, ideally using a flow diagram. | Page 8 and Figure 1 |
|  | 16b | Cite studies that might appear to meet the inclusion criteria, but which were excluded, and explain why they were excluded. | N/A |
| Study characteristics | 17 | Cite each included study and present its characteristics. | Page 8-9 and Table S1 |
| Risk of bias in studies | 18 | Present assessments of risk of bias for each included study. | N/A |
| Results of individual studies | 19 | For all outcomes, present, for each study: (a) summary statistics for each group (where appropriate) and (b) an effect estimate and its precision (e.g. confidence/credible interval), ideally using structured tables or plots. | N/A |
| Results of syntheses | 20a | For each synthesis, briefly summarise the characteristics and risk of bias among contributing studies. | N/A |
|  | 20b | Present results of all statistical syntheses conducted. If meta-analysis was done, present for each the summary estimate and its precision (e.g. confidence/credible interval) and measures of statistical heterogeneity. If comparing groups, describe the direction of the effect. | N/A |
|  | 20c | Present results of all investigations of possible causes of heterogeneity among study results. | N/A |
|  | 20d | Present results of all sensitivity analyses conducted to assess the robustness of the synthesized results. | N/A |
| Reporting biases | 21 | Present assessments of risk of bias due to missing results (arising from reporting biases) for each synthesis assessed. | N/A |
| Certainty of evidence | 22 | Present assessments of certainty (or confidence) in the body of evidence for each outcome assessed. | N/A |
| **DISCUSSION** | | |  |
| Discussion | 23a | Provide a general interpretation of the results in the context of other evidence. | Page 18-24 |
|  | 23b | Discuss any limitations of the evidence included in the review. | Page 18-24 |
|  | 23c | Discuss any limitations of the review processes used. | Page 18-24 |
|  | 23d | Discuss implications of the results for practice, policy, and future research. | Page 23-24 |
| **OTHER INFORMATION** | | |  |
| Registration and protocol | 24a | Provide registration information for the review, including register name and registration number, or state that the review was not registered. | Page 5 |
|  | 24b | Indicate where the review protocol can be accessed, or state that a protocol was not prepared. | Page 5 |
|  | 24c | Describe and explain any amendments to information provided at registration or in the protocol. | Page 22 |
| Support | 25 | Describe sources of financial or non-financial support for the review, and the role of the funders or sponsors in the review. | Page 25 |
| Competing interests | 26 | Declare any competing interests of review authors. | Page 25 |
| Availability of data, code and other materials | 27 | Report which of the following are publicly available and where they can be found: template data collection forms; data extracted from included studies; data used for all analyses; analytic code; any other materials used in the review. | Page 24-25 |

*From:*  Page MJ, McKenzie JE, Bossuyt PM, Boutron I, Hoffmann TC, Mulrow CD, et al. The PRISMA 2020 statement: an updated guideline for reporting systematic reviews. BMJ 2021;372:n71. doi: 10.1136/bmj.n71. This work is licensed under CC BY 4.0. To view a copy of this license, visit <https://creativecommons.org/licenses/by/4.0/>

References

1. Aceves-Martins M, Lopez-Cruz L, Garcia-Botello M, Gutierrez-Gomez YY, Moreno-Garcia CF. Interventions to Prevent Obesity in Mexican Children and Adolescents: Systematic Review. *Prevention Science.* 2022;23(4):563-586.

2. Aceves-Martins M, López-Cruz L, García-Botello M, Gutierrez-Gómez YY, Moreno-García CF. Interventions to Treat Obesity in Mexican Children and Adolescents: Systematic Review and Meta-Analysis. *Nutrition Reviews.* 2022;80(3):544-560.

3. Adom T, De Villiers A, Puoane T, Kengne AP. School-Based Interventions Targeting Nutrition and Physical Activity, and Body Weight Status of African Children: A Systematic Review. *Nutrients.* 2020;12(1):95.

4. Allcott-Watson H, Chater A, Troop N, Howlett N. A systematic review of interventions targeting physical activity and/or healthy eating behaviours in adolescents: Practice and training. *Health Psychology Review.* 2023:No Pagination Specified.

5. Andrade J, Lotton J, Andrade J. Systematic Review: Frameworks Used in School-Based Interventions, the Impact on Hispanic Children's Obesity-Related Outcomes. *Journal of School Health.* 2018;88(11):847-858.

6. Andreo CL, Andrade JM. Determining Effective Nutrition Intervention Strategies and the Subsequent Impact on Nutrition Knowledge, Dietary Adherence, and Health Outcomes among American Indian/Alaska Native Youth (2-18 Years of Age): a Systematic Review. *Journal of Racial and Ethnic Health Disparities.* 2020;7(6):1202-1213.

7. Andueza N, Navas-Carretero S, Cuervo M. Effectiveness of Nutritional Strategies on Improving the Quality of Diet of Children from 6 to 12 Years Old: A Systematic Review. *Nutrients.* 2022;14(2):372-372.

8. Angawi K, Gaissi A. Systematic Review of Setting-Based Interventions for Preventing Childhood Obesity. *BioMed Research International.* 2021:1-10.

9. Bagherniya M, Taghipour A, Sharma M, et al. Obesity intervention programs among adolescents using social cognitive theory: a systematic literature review. *Health Education Research.* 2018;33(1):26-39.

10. Bailey CJ, Drummond MJ, Ward PR. Food literacy programmes in secondary schools: a systematic literature review and narrative synthesis of quantitative and qualitative evidence. *Public Health Nutrition.* 2019;22(15):2891-2913.

11. Bel-Serrat S, Greene E, Mullee A, Murrin CM. Theoretical and practical approaches for dietary behavior change in urban socioeconomically disadvantaged adolescents: a systematic review. *Nutrition Reviews.* 2022;80(6):1531-1557.

12. Bennett AE, Mockler D, Cunningham C, Glennon-Slattery C, Johnston Molloy C. A review of experiential school-based culinary interventions for 5–12-year-old children. *Children.* 2021;8(12):1080.

13. Brown T, Moore THM, Hooper L, et al. Interventions for preventing obesity in children. *Cochrane Database of Systematic Reviews.* 2019(7).

14. Buru K, Emeto TI, Malau-Aduli AEO, Malau-Aduli BS. The Efficacy of School-Based Interventions in Preventing Adolescent Obesity in Australia. *Healthcare.* 2020;8(4).

15. Calvert S, Dempsey RC, Povey R. Delivering in-school interventions to improve dietary behaviours amongst 11-to 16-year-olds: A systematic review. *Obesity Reviews.* 2019;20(4):543-553.

16. Carducci B, Oh C, Keats EC, Roth DE, Bhutta ZA. Effect of Food Environment Interventions on Anthropometric Outcomes in School-Aged Children and Adolescents in Low- and Middle-Income Countries: A Systematic Review and Meta-Analysis. *Current Developments in Nutrition.* 2020;4(7).

17. Cerrato-Carretero P, Roncero-Martin R, Pedrera-Zamorano JD, et al. Long-Term Dietary and Physical Activity Interventions in the School Setting and Their Effects on BMI in Children Aged 6-12 Years: Meta-Analysis of Randomized Controlled Clinical Trials. *Healthcare.* 2021;9(4).

18. Champion KE, Parmenter B, McGowan C, et al. Effectiveness of school-based eHealth interventions to prevent multiple lifestyle risk behaviours among adolescents: a systematic review and meta-analysis. *Lancet Digital Health.* 2019;1(5):E206-E221.

19. Chaudhary A, Sudzina F, Mikkelsen BE. Promoting Healthy Eating among Young People-A Review of the Evidence of the Impact of School-Based Interventions. *Nutrients.* 2020;12(9).

20. Chavez RC, Nam EW. School-based obesity prevention interventions in Latin America: A systematic review. *Revista De Saude Publica.* 2020;54.

21. Cohen JFW, Hecht AA, McLoughlin GM, Turner L, Schwartz MB. Universal School Meals and Associations with Student Participation, Attendance, Academic Performance, Diet Quality, Food Security, and Body Mass Index: A Systematic Review. *Nutrients.* 2021;13(3).

22. Collado-Soler R, Alférez-Pastor M, Torres FL, Trigueros R, Aguilar-Parra JM, Navarro N. A Systematic Review of Healthy Nutrition Intervention Programs in Kindergarten and Primary Education. *Nutrients.* 2023;15(3):541.

23. Colley P, Myer B, Seabrook J, Gilliland J. The Impact of Canadian School Food Programs on Children's Nutrition and Health: A Systematic Review. *Canadian Journal of Dietetic Practice and Research.* 2019;80(2):79-86.

24. Comeau A, Mertens B, Bachwal L, Utter J, van Herwerden L. Effectiveness of nutrition interventions in Australian secondary schools: A systematic review. *Health promotion journal of Australia : official journal of Australian Association of Health Promotion Professionals.* 2023;16.

25. Cotton W, Dudley D, Peralta L, Werkhoven T. The effect of teacher-delivered nutrition education programs on elementary-aged students: An updated systematic review and meta-analysis. *Preventive Medicine Reports.* 2020;20.

26. Dabravolskaj J, Montemurro G, Ekwaru JP, et al. Effectiveness of school-based health promotion interventions prioritized by stakeholders from health and education sectors: A systematic review and meta-analysis. *Preventive Medicine Reports.* 2020;19:101138.

27. Dallagiacoma G, Alberti F, Odone A. The efficacy of digital media tools to promote a healthy diein children: A systematic review of intervention studies. *Acta Biomedica.* 2023;94.

28. de Sousa D, Fogel A, Azevedo J, Padrão P. The Effectiveness of Web-Based Interventions to Promote Health Behaviour Change in Adolescents: A Systematic Review. *Nutrients.* 2022;14(6):1258.

29. Dias RS, Barros AN, Silva AJ, et al. The effect of school intervention programs on the body mass index of adolescents: a systematic review with meta-analysis. *Health Education Research.* 2020;35(5):396-406.

30. Dimple D, Ramesh G. Cooking and Its Impact on Childhood Obesity: A Systematic Review. *Journal of Nutrition Education & Behavior.* 2023;55(9):677-688.

31. Egan L, Gardner LA, Newton N, Champion K. A systematic review of ehealth interventions among adolescents of low socioeconomic and geographically remote backgrounds in preventing poor diet, alcohol use, tobacco smoking and vaping. *Adolescent Research Review.* 2023:No Pagination Specified.

32. Flores-Vazquez AS, Rodriguez-Rocha NP, Herrera-Echauri DD, Macedo-Ojeda G. A systematic review of educational nutrition interventions based on behavioral theories in school adolescents. *Appetite.* 2024;192.

33. Franca C, Santos F, Martins F, et al. Digital Health in Schools: A Systematic Review. *Sustainability.* 2022;14(21).

34. Godoy-Cumillaf A, Fuentes-Merino P, Díaz-González A, et al. The Effects of Physical Activity and Diet Interventions on Body Mass Index in Latin American Children and Adolescents: A Systematic Review and Meta-Analysis. *Nutrients.* 2020;12(5).

35. Gordon K, Dynan L, Siegel R. Healthier Choices in School Cafeterias: A Systematic Review of Cafeteria Interventions. *J Pediatr.* 2018;203:273-279.e272.

36. Harris JA, Carins JE, Rundle-Thiele S. A systematic review of interventions to increase breakfast consumption: a socio-cognitive perspective. *Public Health Nutr.* 2021;24(11):3253-3268.

37. Harrison MR. Effects of public-private partnership on diet-related obesity risk factors among school-aged children: A systematic literature review. *Nutr Health.* 2023;29(3):453-463.

38. Hasan B, Thompson WG, Almasri J, et al. The effect of culinary interventions (cooking classes) on dietary intake and behavioral change: a systematic review and evidence map. *BMC Nutr.* 2019;5:29.

39. Hayba N, Rissel C, Allman Farinelli M. Effectiveness of lifestyle interventions in preventing harmful weight gain among adolescents: A systematic review of systematic reviews. *Obes Rev.* 2021;22(2):e13109.

40. Ho TJH, Cheng LJ, Lau Y. School-based interventions for the treatment of childhood obesity: a systematic review, meta-analysis and meta-regression of cluster randomised controlled trials. *Public Health Nutr.* 2021;24(10):3087-3099.

41. Ismail MR, Seabrook JA, Gilliland JA. Outcome evaluation of fruits and vegetables distribution interventions in schools: a systematic review and meta-analysis. *Public Health Nutr.* 2021;24(14):4693-4705.

42. Jacob CM, Hardy-Johnson PL, Inskip HM, et al. A systematic review and meta-analysis of school-based interventions with health education to reduce body mass index in adolescents aged 10 to 19 years. *Int J Behav Nutr Phys Act.* 2021;18(1):1.

43. Klingberg S, Draper CE, Micklesfield LK, Benjamin-Neelon SE, van Sluijs EMF. Childhood Obesity Prevention in Africa: A Systematic Review of Intervention Effectiveness and Implementation. *Int J Environ Res Public Health.* 2019;16(7).

44. Kyere P, Veerman JL, Lee P, Stewart DE. Effectiveness of school-based nutrition interventions in sub-Saharan Africa: a systematic review. *Public Health Nutr.* 2020;23(14):2626-2636.

45. Lavelle MA, Knopp M, Gunther CW, Hopkins LC. Youth and Peer Mentor Led Interventions to Improve Biometric-, Nutrition, Physical Activity, and Psychosocial-Related Outcomes in Children and Adolescents: A Systematic Review. *Nutrients.* 2023;15(12):07.

46. Leme ACB, Haines J, Tang L, et al. Impact of Strategies for Preventing Obesity and Risk Factors for Eating Disorders among Adolescents: A Systematic Review. *Nutrients.* 2020;12(10):3134.

47. Liu Z, Xu HM, Wen LM, et al. A systematic review and meta-analysis of the overall effects of school-based obesity prevention interventions and effect differences by intervention components. *Int J Behav Nutr Phys Act.* 2019;16(1):95.

48. Long Q, Zhang T, Chen F, Wang W, Chen X, Ma M. Effectiveness of dietary interventions on weight outcomes in childhood: a systematic review meta-analysis of randomized controlled trials. *Transl Pediatr.* 2021;10(4):701-714.

49. Lopez-Gil JF, Victoria-Montesinos D, Garcia-Hermoso A. Effects of lifestyle-based interventions to improve Mediterranean diet adherence among the young population: a meta-analysis of randomized controlled trials. *Nutrition reviews.* 2023;6.

50. Mandracchia F, Tarro L, Llauradó E, Valls RM, Solà R. Interventions to Promote Healthy Meals in Full-Service Restaurants and Canteens: A Systematic Review and Meta-Analysis. *Nutrients.* 2021;13(4).

51. Marcano-Olivier MI, Horne PJ, Viktor S, Erjavec M. Using Nudges to Promote Healthy Food Choices in the School Dining Room: A Systematic Review of Previous Investigations. *J Sch Health.* 2020;90(2):143-157.

52. McHugh C, Hurst A, Bethel A, Lloyd J, Logan S, Wyatt K. The impact of the World Health Organization Health Promoting Schools framework approach on diet and physical activity behaviours of adolescents in secondary schools: a systematic review. *Public Health.* 2020;182:116-124.

53. Medeiros G, Azevedo KPM, Garcia D, et al. Effect of School-Based Food and Nutrition Education Interventions on the Food Consumption of Adolescents: A Systematic Review and Meta-Analysis. *Int J Environ Res Public Health.* 2022;19(17).

54. Metcalfe JJ, Ellison B, Hamdi N, Richardson R, Prescott MP. A systematic review of school meal nudge interventions to improve youth food behaviors. *International Journal of Behavioral Nutrition and Physical Activity.* 2020;17(1):77.

55. Mingay E, Hart M, Yoong S, et al. The Impact of Modifying Food Service Practices in Secondary Schools Providing a Routine Meal Service on Student's Food Behaviours, Health and Dining Experience: A Systematic Review and Meta-Analysis. *Nutrients.* 2022;14(17).

56. Moore SG, Donnelly JK, Jones S, Cade JE. Effect of Educational Interventions on Understanding and Use of Nutrition Labels: A Systematic Review. *Nutrients.* 2018;10(10).

57. Morgan EH, Schoonees A, Sriram U, Faure M, Seguin-Fowler RA. Caregiver involvement in interventions for improving children's dietary intake and physical activity behaviors. *Cochrane Database Syst Rev.* 2020;1(1):Cd012547.

58. Muzaffar H, Guenther E, Bosse O, Nii-Aponsah H. Effectiveness of Gardening-Only, Cooking-Only and Combined Cooking and Gardening Programs in Elementary Schools to Improve Fruit and Vegetable Intake: A Systematic Review. *Nutrients.* 2023;15(13):30.

59. Nakabayashi J, Melo GR, Toral N. Transtheoretical model-based nutritional interventions in adolescents: a systematic review. *BMC Public Health.* 2020;20(1):1543.

60. Nally S, Carlin A, Blackburn NE, et al. The Effectiveness of School-Based Interventions on Obesity-Related Behaviours in Primary School Children: A Systematic Review and Meta-Analysis of Randomised Controlled Trials. *Children (Basel).* 2021;8(6).

61. Nathan N, Janssen L, Sutherland R, et al. The effectiveness of lunchbox interventions on improving the foods and beverages packed and consumed by children at centre-based care or school: a systematic review and meta-analysis. *Int J Behav Nutr Phys Act.* 2019;16(1):38.

62. Navidad L, Padial-Ruz R, González MC. Nutrition, Physical Activity, and New Technology Programs on Obesity Prevention in Primary Education: A Systematic Review. *Int J Environ Res Public Health.* 2021;18(19).

63. Nikniaz Z, Tabrizi JS, Ghojazadeh M, et al. Community-based interventions to increase dairy intake in healthy populations: a systematic review. *Public Health Rev.* 2020;41:18.

64. Nonguierma E, Lesco E, Olak R, et al. Improving Obesogenic Dietary Behaviors among Adolescents: A Systematic Review of Randomized Controlled Trials. *Nutrients.* 2022;14(21).

65. Obita G, Alkhatib A. Effectiveness of Lifestyle Nutrition and Physical Activity Interventions for Childhood Obesity and Associated Comorbidities among Children from Minority Ethnic Groups: A Systematic Review and Meta-Analysis. *Nutrients.* 2023;15(11):29.

66. Oh C, Carducci B, Vaivada T, Bhutta ZA. Digital Interventions for Universal Health Promotion in Children and Adolescents: A Systematic Review. *Pediatrics.* 2022;149(Suppl 5).

67. Olarte DA, Tsai MM, Chapman L, Hager ER, Cohen JFW. Alternative School Breakfast Service Models and Associations with Breakfast Participation, Diet Quality, Body Mass Index, Attendance, Behavior, and Academic Performance: A Systematic Review. *Nutrients.* 2023;15(13):29.

68. Omidvar N, Doustmohammadian A, Shakibazadeh E, Clark CCT, Kasaii MS, Hajigholam-Saryazdi M. Effects of school-based interventions on Food and Nutrition Literacy (FNLIT) in primary-school-age children: a systematic review. *Br J Nutr.* 2022:1-20.

69. Palmer S, Burton-Obanla A, Goon S, et al. Policy, Systems, and Environmental Changes in Child Nutrition Programs: A Systematic Literature Review. *Advances in nutrition.* 2023;14.

70. Pineda E, Bascunan J, Sassi F. Improving the school food environment for the prevention of childhood obesity: What works and what doesn't. *Obes Rev.* 2021;22(2):e13176.

71. Pongutta S, Ajetunmobi O, Davey C, Ferguson E, Lin L. Impacts of School Nutrition Interventions on the Nutritional Status of School-Aged Children in Asia: A Systematic Review and Meta-Analysis. *Nutrients.* 2022;14(3):589.

72. Prescott MP, Cleary R, Bonanno A, Costanigro M, Jablonski BBR, Long AB. Farm to School Activities and Student Outcomes: A Systematic Review. *Adv Nutr.* 2020;11(2):357-374.

73. Pursey KM, Burrows TL, Barker D, Hart M, Paxton SJ. Disordered eating, body image concerns, and weight control behaviors in primary school aged children: A systematic review and meta-analysis of universal-selective prevention interventions. *Int J Eat Disord.* 2021;54(10):1730-1765.

74. Qi Y, Hamzah SH, Gu E, et al. Is School Gardening Combined with Physical Activity Intervention Effective for Improving Childhood Obesity? A Systematic Review and Meta-Analysis. *Nutrients.* 2021;13(8).

75. Abdel Rahman A, Jomaa L, Kahale LA, Adair P, Pine C. Effectiveness of behavioral interventions to reduce the intake of sugar-sweetened beverages in children and adolescents: a systematic review and meta-analysis. *Nutr Rev.* 2018;76(2):88-107.

76. Rose K, O'Malley C, Eskandari F, Lake AA, Brown L, Ells LJ. The impact of, and views on, school food intervention and policy in young people aged 11-18 years in Europe: A mixed methods systematic review. *Obes Rev.* 2021;22(5):e13186.

77. Salam RA, Das JK, Ahmed W, Irfan O, Sheikh SS, Bhutta ZA. Effects of Preventive Nutrition Interventions among Adolescents on Health and Nutritional Status in Low- and Middle-Income Countries: A Systematic Review and Meta-Analysis. *Nutrients.* 2019;12(1).

78. Schulte EA, Winkler G, Brombach C, Buyken AE. Choice architecture interventions promoting sustained healthier food choice and consumption by students in a secondary school setting: a systematic review of intervention studies. *Public Health Nutrition.* 2023;26(9):1896-1906.

79. Seral-Cortes M, De Miguel-Etayo P, Zapata P, Miguel-Berges ML, Moreno LA. Effectiveness and process evaluation in obesity and type 2 diabetes prevention programs in children: a systematic review and meta-analysis. *BMC Public Health.* 2021;21(1):348.

80. Charles Shapu R, Ismail S, Ahmad N, Lim PY, Abubakar Njodi I. Systematic Review: Effect of Health Education Intervention on Improving Knowledge, Attitudes and Practices of Adolescents on Malnutrition. *Nutrients.* 2020;12(8).

81. Abu Shihab KHN, Dodge E, Benajiba N, Chavarria EA, Aboul-Enein BH, Faris MAE. Effectiveness of school-based nutrition interventions promoted in the League of Arab States: a systematic review. *Health Promotion International.* 2023;38(4):01.

82. Beck Silva KB, Miranda Pereira E, Santana MLPD, Costa PRF, Silva RDCR. Effects of computer-based interventions on food consumption and anthropometric parameters of adolescents: A systematic review and metanalysis. *Critical Reviews in Food Science and Nutrition.* 2022.

83. Singhal J, Herd C, Adab P, Pallan M. Effectiveness of school‐based interventions to prevent obesity among children aged 4 to 12 years old in middle‐income countries: a systematic review and meta‐analysis. *Obesity reviews.* 2021;22(1):e13105.

84. Smit MS, Boelens M, Mölenberg FJM, Raat H, Jansen W. The long-term effects of primary school-based obesity prevention interventions in children: A systematic review and meta-analysis. *Pediatric Obesity.* 2023;18(3):e12997.

85. Smith C, Goss HR, Issartel J, Belton S. Health Literacy in Schools? A Systematic Review of Health-Related Interventions Aimed at Disadvantaged Adolescents. *Children (Basel).* 2021;8(3).

86. Sutherland RL, Jackson JK, Lane C, et al. A systematic review of adaptations and effectiveness of scaled-up nutrition interventions. *Nutr Rev.* 2022;80(4):962-979.

87. Taghizadeh S, Farhangi MA. The effectiveness of pediatric obesity prevention policies: a comprehensive systematic review and dose-response meta-analysis of controlled clinical trials. *J Transl Med.* 2020;18(1):480.

88. Tallon JM, Saavedra Dias R, Costa AM, et al. Impact of Technology and School-Based Nutrition Education Programs on Nutrition Knowledge and Behavior During Adolescence—A Systematic Review. *Scandinavian Journal of Educational Research.* 2021;65(1):169-180.

89. Varman SD, Cliff DP, Jones RA, et al. Experiential Learning Interventions and Healthy Eating Outcomes in Children: A Systematic Literature Review. *Int J Environ Res Public Health.* 2021;18(20).

90. Vega-Salas MJ, Murray C, Nunes R, et al. School environments and obesity: a systematic review of interventions and policies among school-age students in Latin America and the Caribbean. *Int J Obes (Lond).* 2023;47(1):5-16.

91. Verjans-Janssen SRB, van de Kolk I, Van Kann DHH, Kremers SPJ, Gerards S. Effectiveness of school-based physical activity and nutrition interventions with direct parental involvement on children's BMI and energy balance-related behaviors - A systematic review. *PLoS One.* 2018;13(9):e0204560.

92. von Philipsborn P, Stratil JM, Burns J, et al. Environmental interventions to reduce the consumption of sugar-sweetened beverages and their effects on health. *Cochrane Database Syst Rev.* 2019;6(6):Cd012292.

93. Wahi G, de Souza RJ, Hartmann K, Giglia L, Jack SM, Anand SS. Effectiveness of programs aimed at obesity prevention among Indigenous children: A systematic review. *Prev Med Rep.* 2021;22:101347.

94. Wall C, Tolar-Peterson T, Reeder N, Roberts M, Reynolds A, Rico Mendez G. The Impact of School Meal Programs on Educational Outcomes in African Schoolchildren: A Systematic Review. *Int J Environ Res Public Health.* 2022;19(6).

95. Wang D, Shinde S, Young T, Fawzi WW. Impacts of school feeding on educational and health outcomes of school-age children and adolescents in low- and middle-income countries: A systematic review and meta-analysis. *J Glob Health.* 2021;11:04051.

96. Whitehead L, Kabdebo I, Dunham M, et al. The effectiveness of nurse-led interventions to prevent childhood and adolescent overweight and obesity: A systematic review of randomised trials. *J Adv Nurs.* 2021;77(12):4612-4631.

97. Xu T, Tomokawa S, Gregorio ER, Jr., Mannava P, Nagai M, Sobel H. School-based interventions to promote adolescent health: A systematic review in low- and middle-income countries of WHO Western Pacific Region. *PLoS One.* 2020;15(3):e0230046.

98. Zuair AA, Sopory P. Effects of Media Health Literacy School-Based Interventions on Adolescents' Body Image Concerns, Eating Concerns, and Thin-Internalization Attitudes: A Systematic Review and Meta-Analysis. *Health Commun.* 2022;37(1):20-28.

99. Cade JE, Frear L, Greenwood DC. Assessment of diet in young children with an emphasis on fruit and vegetable intake: using CADET--Child and Diet Evaluation Tool. *Public Health Nutr.* 2006;9(4):501-508.

100. Christian M, Evans C, Nykjaer C, Hancock N, Cade J. Measuring diet in primary school children aged 8-11 years: validation of the Child and Diet Evaluation Tool (CADET) with an emphasis on fruit and vegetable intake. *European journal of clinical nutrition.* 2015;69(2):234-241.

101. Edmunds L, Ziebland S. Development and validation of the Day in the Life Questionnaire (DILQ) as a measure of fruit and vegetable questionnaire for 7–9 year olds. *Health education research.* 2002;17(2):211-220.

102. Hunsberger M, O'Malley J, Block T, Norris JC. Relative validation of B lock K ids F ood S creener for dietary assessment in children and adolescents. *Maternal & child nutrition.* 2015;11(2):260-270.

103. Rockett HR, Wolf AM, Colditz GA. Development and reproducibility of a food frequency questionnaire to assess diets of older children and adolescents. *Journal of the American Dietetic Association.* 1995;95(3):336-340.

104. Rockett HR, Breitenbach M, Frazier AL, et al. Validation of a youth/adolescent food frequency questionnaire. *Prev Med.* 1997;26(6):808-816.

105. Buzzard IM, Stanton CA, Figueiredo M, et al. Development and reproducibility of a brief food frequency questionnaire for assessing the fat, fiber, and fruit and vegetable intakes of rural adolescents. *Journal of the American Dietetic Association.* 2001;101(12):1438-1446.

106. Casazza K, Ciccazzo M. The method of delivery of nutrition and physical activity information may play a role in eliciting behavior changes in adolescents. *Eating Behaviors.* 2007;8(1):73-82.

107. Watson JF, Collins CE, Sibbritt DW, Dibley MJ, Garg ML. Reproducibility and comparative validity of a food frequency questionnaire for Australian children and adolescents. *International Journal of Behavioral Nutrition and Physical Activity.* 2009;6:1-17.

108. Raby Powers A, Struempler BJ, Guarino A, Parmer SM. Effects of a nutrition education program on the dietary behavior and nutrition knowledge of second‐grade and third‐grade students. *Journal of school health.* 2005;75(4):129-133.

109. Birnbaum AS, Lytle LA, Story M, Perry CL, Murray DM. Are differences in exposure to a multicomponent school-based intervention associated with varying dietary outcomes in adolescents? *Health Education & Behavior.* 2002;29(4):427-443.

110. Serra-Majem L, Ribas L, Ngo J, et al. Food, youth and the Mediterranean diet in Spain. Development of KIDMED, Mediterranean Diet Quality Index in children and adolescents. *Public Health Nutr.* 2004;7(7):931-935.

111. Aparicio-Ugarriza R, Cuenca-García M, Gonzalez-Gross M, et al. Relative validation of the adapted Mediterranean Diet Score for Adolescents by comparison with nutritional biomarkers and nutrient and food intakes: The Healthy Lifestyle in Europe by Nutrition in Adolescence (HELENA) study. *Public Health Nutrition.* 2019;22(13):2381-2397.

112. Keshani P, Hossein Kaveh M, Faghih S, Salehi M. Improving diet quality among adolescents, using health belief model in a collaborative learning context: a randomized field trial study. *Health Education Research.* 2019;34(3):279-288.

113. Sabinsky MS, Toft U, Sommer HM, Tetens I. Effect of implementing school meals compared with packed lunches on quality of dietary intake among children aged 7-13 years. *J Nutr Sci.* 2019;8:e3.

114. Sabinsky MS, Toft U, Andersen KK, Tetens I. Development and validation of a Meal Index of dietary Quality (Meal IQ) to assess the dietary quality of school lunches. *Public Health Nutr.* 2012;15(11):2091-2099.

115. Lassen AD, Biltoft-Jensen A, Hansen GL, Hels O, Tetens I. Development and validation of a new simple Healthy Meal Index for canteen meals. *Public Health Nutrition.* 2010;13(10):1559-1565.

116. Mack I, Reiband N, Etges C, et al. The Kids Obesity Prevention Program: Cluster Randomized Controlled Trial to Evaluate a Serious Game for the Prevention and Treatment of Childhood Obesity. *J Med Internet Res.* 2020;22(4):e15725.

117. Teo CH, Chin YS, Lim PY, Masrom SAH, Shariff ZM. Impacts of a School-Based Intervention That Incorporates Nutrition Education and a Supportive Healthy School Canteen Environment among Primary School Children in Malaysia. *Nutrients.* 2021;13(5).

118. Frenn M, Malin S, Brown RL, et al. Changing the tide: an Internet/video exercise and low-fat diet intervention with middle-school students. *Applied nursing research.* 2005;18(1):13-21.

119. Frenn M, Malin S, Bansal NK. Stage-based interventions for low-fat diet with middle school students. *Journal of Pediatric Nursing.* 2003;18(1):36-45.

120. Greene GW, Resnicow K, Thompson FE, et al. Correspondence of the NCI Fruit and Vegetable Screener to repeat 24-H recalls and serum carotenoids in behavioral intervention trials. *The Journal of nutrition.* 2008;138(1):200S-204S.

121. Gustafson A, Jilcott Pitts SB, McQuerry K, Babtunde O, Mullins J. A mentor-led text-messaging intervention increases intake of fruits and vegetables and goal setting for healthier dietary consumption among rural adolescents in Kentucky and North Carolina, 2017. *Nutrients.* 2019;11(3):593.

122. Hedrick VE, Savla J, Comber DL, et al. Development of a brief questionnaire to assess habitual beverage intake (BEVQ-15): sugar-sweetened beverages and total beverage energy intake. *Journal of the Academy of Nutrition and Dietetics.* 2012;112(6):840-849.

123. Biltoft-Jensen A, Bysted A, Trolle E, et al. Evaluation of Web-based Dietary Assessment Software for Children: comparing reported fruit, juice and vegetable intakes with plasma carotenoid concentration and school lunch observations. *British journal of nutrition.* 2013;110(1):186-195.

124. Lee JE, Song S, Ahn JS, Kim Y, Lee JE. Use of a Mobile Application for Self-Monitoring Dietary Intake: Feasibility Test and an Intervention Study. *Nutrients.* 2017;9(7).

125. Yang Y-TC, Wang C-J, Tsai M-F, Wang J-S. Technology-enhanced game-based team learning for improving intake of food groups and nutritional elements. *Computers & Education.* 2015;88:143-159.

126. Bennett C, Blissett J. Measuring hunger and satiety in primary school children. Validation of a new picture rating scale. *Appetite.* 2014;78:40-48.

127. Kleinman RE, Hall S, Green H, et al. Diet, breakfast, and academic performance in children. *Annals of Nutrition and Metabolism.* 2002;46(Suppl. 1):24-30.

128. Mhurchu CN, Gorton D, Turley M, et al. Effects of a free school breakfast programme on children's attendance, academic achievement and short-term hunger: results from a stepped-wedge, cluster randomised controlled trial. *J Epidemiol Community Health.* 2013;67(3):257-264.

129. Kleinman RE, Murphy JM, Little M, et al. Hunger in children in the United States: potential behavioral and emotional correlates. *Pediatrics.* 1998;101(1):e3-e3.

130. Dalma A, Petralias A, Tsiampalis T, et al. Effectiveness of a school food aid programme in improving household food insecurity; a cluster randomized trial. *European Journal of Public Health.* 2020;30(1):171-178.

131. Petralias A, Papadimitriou E, Riza E, Karagas MR, Zagouras AB, Linos A. The impact of a school food aid program on household food insecurity. *Eur J Public Health.* 2016;26(2):290-296.

132. Espinosa-Curiel IE, Pozas-Bogarin EE, Lozano-Salas JL, Martínez-Miranda J, Delgado-Pérez EE, Estrada-Zamarron LS. Nutritional education and promotion of healthy eating behaviors among Mexican children through video games: design and pilot test of FoodRateMaster. *JMIR Serious Games.* 2020;8(2):e16431.

133. Dawson EE. *Development of an Education Curriculum and a Self-Efficacy Scale for Dairy Nutrition in Adolescents*, Bowling Green State University; 2006.

134. Day ME, Strange KS, McKay HA, Naylor P-J. Action schools! BC—healthy eating: effects of a whole-school model to modifying eating behaviours of elementary school children. *Canadian Journal of Public Health.* 2008;99:328-331.

135. Zhou J. Results from a Quasi-Experimental Study of a Training Intervention for Healthy Eating Behaviors of Students *NeuroQuantology.* 2018;16(2):19.

136. Zhou WJ, Xu XL, Li G, Sharma M, Qie YL, Zhao Y. Effectiveness of a school-based nutrition and food safety education program among primary and junior high school students in Chongqing, China. *Glob Health Promot.* 2016;23(1):37-49.

137. Lo E, Coles R, Humbert ML, Polowski J, Henry CJ, Whiting SJ. Beverage intake improvement by high school students in Saskatchewan, Canada. *Nutr Res.* 2008;28(3):144-150.

138. Friel S, Kelleher C, Campbell P, Nolan G. Evaluation of the nutrition education at primary school (NEAPS) programme. *Public health nutrition.* 1999;2(4):549-555.

139. Gates M, Hanning RM, Gates A, Isogai A, Tsuji LJ, Metatawabin J. A pilot comprehensive school nutrition program improves knowledge and intentions for intake of milk and milk alternatives among youth in a remote First Nation. *Journal of nutrition education and behavior.* 2013;45(5):455-459.

140. Gates A, Hanning R, Gates M, Isogai A, Metatawabin J, Tsuji L. A school nutrition program improves vegetable and fruit knowledge, preferences, and exposure in First Nation youth. *The Open Nutrition Journal.* 2011;5(1).

141. Skinner K, Hanning RM, Metatawabin J, Martin ID, Tsuji LJ. Impact of a school snack program on the dietary intake of grade six to ten First Nation students living in a remote community in northern Ontario, Canada. *Rural Remote Health.* 2012;12:2122.

142. Gavaravarapu SM, Saha S, Vemula SR, Mendu VVR. Read-B4-U-Eat: A multicomponent communication module to promote food label reading skills among adolescents in India. *Journal of nutrition education and behavior.* 2016;48(8):586-589. e581.

143. Ratcliffe MM, Merrigan KA, Rogers BL, Goldberg JP. The effects of school garden experiences on middle school-aged students' knowledge, attitudes, and behaviors associated with vegetable consumption. *Health Promot Pract.* 2011;12(1):36-43.

144. Katz DL, Treu JA, Ayettey RG, Kavak Y, Katz CS, Njike V. Testing the effectiveness of an abbreviated version of the Nutrition Detectives program. *Prev Chronic Dis.* 2014;11:E57.

145. Currie C, Samdal O, Boyce W, Smith R. Health behaviour in school-aged children: a WHO cross-national study (HBSC), research protocol for the 2001/2002 survey. *Child and Adolescent Health Research Unit (CAHRU), University of Edinburgh.* 2001;20.

146. Busch V, De Leeuw JRJ, Zuithoff NP, Van Yperen TA, Schrijvers AJP. A controlled health promoting school study in the Netherlands: effects after 1 and 2 years of intervention. *Health promotion practice.* 2015;16(4):592-600.

147. Parcel GS, Edmundson E, Perry CL, et al. Measurement of self-efficacy for diet-related behaviors among elementary school children. *J Sch Health.* 1995;65(1):23-27.

148. Grey M, Jaser SS, Holl MG, Jefferson V, Dziura J, Northrup V. A multifaceted school-based intervention to reduce risk for type 2 diabetes in at-risk youth. *Preventive medicine.* 2009;49(2-3):122-128.

149. Brener ND, Collins JL, Kann L, Warren CW, Williams BI. Reliability of the youth risk behavior survey questionnaire. *American journal of epidemiology.* 1995;141(6):575-580.

150. Malakellis M, Hoare E, Sanigorski A, et al. School-based systems change for obesity prevention in adolescents: outcomes of the Australian Capital Territory 'It's Your Move!'. *Aust N Z J Public Health.* 2017;41(5):490-496.

151. Lynch BA, Gentile N, Maxson J, Quigg S, Swenson L, Kaufman T. Elementary School-Based Obesity Intervention Using an Educational Curriculum. *J Prim Care Community Health.* 2016;7(4):265-271.

152. Brown KW, Ryan RM. Mindful attention awareness scale. *Journal of personality and social psychology.* 2003.

153. Lawlor MS, Schonert-Reichl KA, Gadermann AM, Zumbo BD. A validation study of the mindful attention awareness scale adapted for children. *Mindfulness.* 2014;5:730-741.

154. Kovacs M. Children’s Depression Inventory. *CDI, Manual/Multi-Health Systems Inc.* 1992.

155. Timbremont B, Braet C, Dreessen L. Assessing depression in youth: relation between the Children's Depression Inventory and a structured interview. *Journal of Clinical Child and Adolescent Psychology.* 2004;33(1):149-157.

156. Angold A, Costello EJ, Messer SC, Pickles A. Development of a short questionnaire for use in epidemiological studies of depression in children and adolescents. *International Journal of Methods in Psychiatric Research.* 1995;5(4):237–249.

157. Thabrew H, Stasiak K, Bavin LM, Frampton C, Merry S. Validation of the Mood and Feelings Questionnaire (MFQ) and Short Mood and Feelings Questionnaire (SMFQ) in New Zealand help-seeking adolescents. *Int J Methods Psychiatr Res.* 2018;27(3):e1610.

158. Goodman R. The Strengths and Difficulties Questionnaire: a research note. *Journal of child psychology and psychiatry.* 1997;38(5):581-586.

159. Varni JW, Seid M, Kurtin PS. PedsQL 4.0: reliability and validity of the Pediatric Quality of Life Inventory version 4.0 generic core scales in healthy and patient populations. *Med Care.* 2001;39(8):800-812.

160. *The KIDSCREEN questionnaires: Quality of life questionnaires for children and adolescents.* Lengerich, Germany: Pabst Science Publishers; 2006.

161. van Strien T, Frijters JER, Bergers GPA, Defares PB. The Dutch Eating Behavior Questionnaire (DEBQ) for assessment of restrained, emotional, and external eating behavior. *International Journal of Eating Disorders.* 1986;5(2):295-315.

162. Garner DM, Olmsted MP, Bohr Y, Garfinkel PE. The eating attitudes test: psychometric features and clinical correlates. *Psychological medicine.* 1982;12(4):871-878.

163. Garner DM, Garfinkel PE. The Eating Attitudes Test: An index of the symptoms of anorexia nervosa. *Psychological medicine.* 1979;9(2):273-279.

164. Maloney MJ, McGuire JB, Daniels SR. Reliability testing of a children's version of the Eating Attitude Test. *J Am Acad Child Adolesc Psychiatry.* 1988;27(5):541-543.

165. Garner DM, Olmstead MP, Polivy J. Development and validation of a multidimensional eating disorder inventory for anorexia nervosa and bulimia. *International journal of eating disorders.* 1983;2(2):15-34.

166. Scime M, Cook-Cottone C. Primary prevention of eating disorders: a constructivist integration of mind and body strategies. *Int J Eat Disord.* 2008;41(2):134-142.

167. Túry F, Güleç H, Kohls E. Assessment methods for eating disorders and body image disorders. *J Psychosom Res.* 2010;69(6):601-611.

168. Childress AC, Brewerton TD, Hodges EL, Jarrell MP. The Kids' Eating Disorders Survey (KEDS): a study of middle school students. *J Am Acad Child Adolesc Psychiatry.* 1993;32(4):843-850.

169. Mendelson BK, Mendelson MJ, White DR. Body-Esteem Scale for Adolescents and Adults. *Journal of Personality Assessment.* 2001;76(1):90-106.

170. McCabe MP, Connaughton C, Tatangelo G, Mellor D, Busija L. Healthy me: A gender-specific program to address body image concerns and risk factors among preadolescents. *Body Image.* 2017;20:20-30.

171. Ricciardelli LA, McCabe MP. Psychometric evaluation of the Body Change Inventory: an assessment instrument for adolescent boys and girls. *Eat Behav.* 2002;3(1):45-59.

172. McCabe MP, Ricciardelli LA. Sociocultural influences on body image and body changes among adolescent boys and girls. *The Journal of social psychology.* 2003;143(1):5-26.

173. Smolak L, Stein J. Male Physical Attributes Investment Scale. Unpublished scale. . 2005.

174. Downes J, Evenden J, Morris R, et al. Cambridge Neurological Test Automated Battery (CANTAB): Instruction Manual. *Waterbeach, UK: Paul Fray Ltd.* 1994.

175. Wechsler D, Kodama H. *Wechsler intelligence scale for children.* Vol 1: Psychological corporation New York; 1949.

176. Eigsti I-M. Peabody picture vocabulary test. In: *Encyclopedia of Autism Spectrum Disorders.* Springer; 2021:3357-3360.

177. Bere E, Veierød M, Bjelland M, Klepp K. Outcome and process evaluation of a Norwegian school-randomized fruit and vegetable intervention: Fruits and Vegetables Make the Marks (FVMM). *Health education research.* 2006;21(2):258-267.
